# Phytosterol-Rich *Delonix regia* Seed Oil Exhibits Antimicrobial, Antibiofilm, Radical-Scavenging and Pancreatic Lipase-Inhibitory Activities: Integrated GC-MS Profiling and Molecular Docking of Stigmasterol

**DOI:** 10.3390/antiox15101249

**Published:** 2026-09-28

**Authors:** Husam Qanash, Aisha M. H. Al-Rajhi, Abdulrahman S. Bazaid, Fahad Almarshadi, Walid Alesefir, Abdu Aldarhami, Waleed Hakami, Amro Duhduh, Abeer Omar Ahmed

**Affiliations:** 1 Department of Medical Laboratory Science, College of Applied Medical Sciences, University of Ha’il, Hail 55476, Saudi Arabia; 2 Medical and Diagnostic Research Center, University of Ha’il, Hail 55473, Saudi Arabia; 3 Department of Biology, College of Science, Princess Nourah bint Abdulrahman University, P.O. Box 84428, Riyadh 11671, Saudi Arabia; 4 Public Health Department, College of Public Health and Health Informatics, University of Ha’il, Hail 55473, Saudi Arabia; 5 Department of Internal Medicine, College of Medicine, University of Ha’il, Hail 55473, Saudi Arabia; 6 Department of Microbiology and Parasitology, Faculty of Medicine, Umm Al-Qura University, Al-Qunfudah 28815, Saudi Arabia; 7 Department of Medical Laboratory Technology, College of Nursing and Health Sciences, Jazan University, Jazan 45142, Saudi Arabia; 8 Department of Pharmaceutics, College of Pharmacy, Jazan University, Jazan 45142, Saudi Arabia

**Keywords:** *Delonix regia*, seed oil, GC-MS, phytosterols, antioxidant activity, antimicrobial activity, antibiofilm activity, pancreatic lipase, stigmasterol, molecular docking

## Abstract

Natural products may address infection, oxidative stress and metabolic disorders. This study used gas chromatography-mass spectrometry (GC-MS), bioassays and molecular docking to characterize *Delonix regia* seed oil and evaluate its antimicrobial, antibiofilm, radical-scavenging and pancreatic lipase-inhibitory activities. The most abundant tentatively identified peaks in the GC-MS total ion chromatogram were β-sitosterol (26.35%), stigmasterol (14.57%), linoleic acid (10.39%), and oleic acid (5.82%). Minimum inhibitory concentrations (MICs) were 15.62 µg/mL for *Listeria monocytogenes*, *Salmonella typhi*, and *Candida albicans*, and 62.5 µg/mL for methicillin-resistant *Staphylococcus aureus*. The corresponding inhibition zones were 28 ± 0.7, 21 ± 0.3, 31 ± 0.1, and 15 ± 0.5 mm, respectively. At 75% of the minimum bactericidal/fungicidal concentration, the oil inhibited biofilm formation by 94.95% for both *L. monocytogenes* and *C. albicans*. DPPH and ABTS radical-scavenging IC_50_ values were 17.04 ± 0.25 and 13.17 ± 0.40 µg/mL, respectively, while the pancreatic lipase IC_50_ was 26.29 ± 0.08 µg/mL. Time-kill assays demonstrated ≥3 log_10_ CFU/mL reductions, with no recoverable colonies for any of the five tested pathogens within 3 h. Docking predicted stronger stigmasterol binding to *L. monocytogenes* Sortase A (−6.60 kcal/mol) than to *C. albicans* lanosterol 14α-demethylase (CYP51; −3.41 kcal/mol), involving hydrogen bonds with Met63 and Arg381, respectively, and hydrophobic contacts. These findings identify a phytosterol-enriched GC-MS-detectable fraction of *Delonix regia* seed oil with in vitro activities warranting further mechanistic and in vivo investigation.

## 1. Introduction

Antimicrobial resistance (AMR), biofilm-associated infections, and diseases linked to oxidative and metabolic imbalance represent interconnected challenges to global health [1,2]. Bacterial AMR alone was associated with an estimated 4.95 million deaths worldwide in 2019 and has been recognized by the World Health Organization as one of the leading threats to public health [3]. The clinical burden is further complicated by the ability of microorganisms to form biofilms, structured communities that can exhibit up to a 1000-fold greater tolerance to conventional antimicrobial agents than their planktonic counterparts [4,5]. These protective communities contribute to persistent and device-associated infections and frequently compromise treatment outcomes. Consequently, there is continued interest in biologically active compounds that can target microbial growth and biofilm formation while offering complementary antioxidant or metabolic effects. Natural products remain an important source of such compounds because of their structural diversity and broad range of biological properties [6,7].

Members of the Fabaceae family are well-established sources of pharmacologically active metabolites [8,9]. *Delonix regia* (Bojer ex Hook.) Raf. (Fabaceae), synonym *Poinciana regia* Bojer ex Hook., commonly known as royal poinciana, is a tropical ornamental tree native to Madagascar and now widely cultivated throughout tropical and subtropical regions. Different parts of the plant have been used traditionally to manage inflammatory conditions, microbial infections, and gastrointestinal disorders [10]. Its seeds are a source of oil containing unsaturated fatty acids, phytosterols, and other lipid-soluble constituents with potential biological value. Previous analyses have identified linoleic and oleic acids as prominent components of seed oil, with stearic acid representing an important saturated fatty acid; however, the reported proportions vary according to geographical origin and analytical conditions [11,12].

Chemical profiling can identify the principal constituents of a natural product but does not, on its own, explain which compounds may contribute to the observed biological effects. Molecular docking provides a complementary means of predicting ligand orientation, relative binding strength, and interactions within selected protein-binding sites [9,13]. In natural-product research, this approach can help prioritize compounds for subsequent experimental assessment and generate testable hypotheses regarding their possible molecular targets [14,15]. Docking predictions should not be regarded as direct evidence of target inhibition; rather, their value lies in supporting the interpretation of experimental findings and guiding mechanistic validation. When combined with gas chromatography-mass spectrometry (GC-MS) and biological assays, docking can provide a useful framework for examining potential relationships between chemical structure and activities involving microbial survival, adhesion, biofilm formation, and metabolic enzymes [16].

Previous investigations have reported antimicrobial and antioxidant activities in extracts prepared from different parts of *D. regia* [17]. However, the seed oil represents a chemically distinct lipophilic fraction that remains considerably less investigated than conventional polar or semipolar plant extracts. Seed oils can contain diverse lipid-soluble constituents, including phytosterols, unsaturated fatty acids, fatty alcohols, and other minor components that may contribute individually or collectively to biological activity. In addition, *D. regia* seeds represent an abundant and relatively underutilized plant resource, providing further rationale for investigating their oil as a potentially bioactive fraction. To our knowledge, no previous study has specifically evaluated *D. regia* seed oil using an integrated approach combining GC-MS chemical profiling with antimicrobial susceptibility testing, biofilm inhibition, time-kill kinetics, radical-scavenging activity, pancreatic lipase inhibition, and pathogen-relevant molecular docking. The present study therefore aimed to characterize the GC-MS-detectable chemical profile of *D. regia* seed oil and evaluate its antimicrobial, antibiofilm, antioxidant, and pancreatic lipase-inhibitory activities. Time-kill analysis was included to assess the rate and extent of microbial viability reduction. In parallel, stigmasterol, one of the most abundant phytosterols tentatively identified in the GC-MS-detectable fraction of the oil, was docked against Sortase A from *Listeria monocytogenes* and lanosterol 14α-demethylase (CYP51) from *Candida albicans*. By integrating chemical, microbiological, antioxidant, enzyme-inhibition, and computational analyses, this study sought to define the bioactivity profile of *D. regia* seed oil and provide a rational basis for subsequent bioassay-guided fractionation and mechanistic validation. Thus, the specific research gap is not the complete absence of previous chemical or biological information on *D. regia*, but rather the lack of a comprehensive evaluation focused specifically on its seed oil and linking its tentatively characterized GC-MS-detectable constituents with multiple biological activities relevant to microbial control, oxidative processes, and metabolic enzyme inhibition.

## 2. Materials and Methods

### 2.1. Plant Material and Oil Extraction

Seeds of *Delonix regia* were collected from mature trees growing in the Hail region of Saudi Arabia during the fruiting season. The collection and experimental use of the plant material complied with applicable institutional and national regulations in the Kingdom of Saudi Arabia and with relevant international guidelines governing plant research. The study was also conducted in accordance with the principles of the Convention on Biological Diversity and the Convention on International Trade in Endangered Species of Wild Fauna and Flora. The plant material was authenticated by a qualified botanist at the Medical and Diagnostic Research Center, University of Ha’il, Saudi Arabia. A voucher specimen was deposited in the center’s herbarium under accession number UOH-MDRC-DR-2026-001. The seeds were manually separated from the pods, air-dried at ambient temperature (25 ± 2 °C) for 7 days, and ground into a fine powder using an electric mill. Seed powder (500 g) was extracted with *n*-hexane at a solid-to-solvent ratio of 1:5 (*w*/*v*) using a Soxhlet apparatus for 8 h at 60 °C. The solvent was removed under reduced pressure using a rotary evaporator at 40 °C, yielding a pale-yellow oil with an extraction yield of 8.2% (*w*/*w*) [18]. The oil was transferred to amber glass vials, flushed with nitrogen to facilitate removal of residual solvent vapor, and stored at −20 °C until analysis. Complete removal of *n*-hexane was not independently verified by headspace GC or another dedicated residual-solvent method. No chromatographic peak attributable to *n*-hexane was identified among the tentatively assigned GC-MS constituents; however, trace residual solvent cannot be excluded without dedicated residual-solvent analysis. Because the oil was analyzed by direct GC-MS without derivatization, the method was intended to profile GC-amenable constituents and was not specifically validated for residual-solvent determination.

### 2.2. Gas Chromatography-Mass Spectrometry (GC-MS) Analysis

The chemical composition of *D. regia* seed oil was analyzed using an Agilent 7890B gas chromatograph (Agilent Technologies Inc., Santa Clara, CA, USA) coupled to an Agilent 5977B mass-selective detector (Agilent Technologies Inc., Santa Clara, CA, USA). Compounds were separated on an HP-5MS capillary column (30 m × 0.25 mm internal diameter; 0.25 µm film thickness). The oven temperature was initially maintained at 50 °C for 2 min, increased to 280 °C at 5 °C/min, and held at 280 °C for 10 min. The injector temperature was set at 250 °C, and helium was used as the carrier gas at a constant flow rate of 1.0 mL/min. A 1 µL aliquot of the oil sample was injected in splitless mode. Mass spectra were acquired under electron-impact ionization at 70 eV over an *m*/*z* range of 40–550. Compounds were tentatively identified by comparing their mass spectra with entries in the National Institute of Standards and Technology (NIST) 2017 spectral library and by comparing their retention indices with published data. For each tentatively identified compound, the NIST match factor and characteristic mass-spectral ions, including the molecular ion and major fragment ions (*m*/*z*), are reported in Table 1. No authentic reference standards were analyzed; therefore, all compound assignments are provisional, and the NIST match factors should be interpreted as spectral-similarity measures rather than evidence of definitive identification. The relative abundance of each compound was calculated from its total-ion chromatogram peak area and expressed as a percentage of the total integrated peak area [19]. This analysis was qualitative and semi-quantitative. No internal standard, calibration curve, authentic reference standard, or response-factor correction was used. The oil was analyzed directly without transesterification or derivatization. Consequently, intact triglycerides and esterified fatty acids are not adequately represented, and the relative TIC peak-area percentages may be biased toward compounds that are more amenable to direct GC-MS analysis. These values therefore represent relative abundances within the GC-MS-detectable fraction and should not be interpreted as absolute concentrations or as the true mass composition of the oil.

### 2.3. Microbial Strains, Culture Conditions and Agar Well-Diffusion Assay

The reference microorganisms used in this study were *Listeria monocytogenes* ATCC 19115, methicillin-resistant *Staphylococcus aureus* ATCC 33591, *Escherichia coli* ATCC 8739, *Salmonella typhi* ATCC 6539, and *Candida albicans* ATCC 10221. Bacterial strains were cultured on Mueller-Hinton agar at 37 °C for 18–24 h, whereas *C. albicans* was cultured on Sabouraud dextrose agar at 30 °C for 48 h. Inocula were prepared by suspending isolated colonies in sterile saline and adjusting the turbidity to a 0.5 McFarland standard, corresponding to approximately 1–2 × 10^8^ colony-forming units (CFU)/mL. Antimicrobial activity was assessed using an agar well-diffusion method adapted from the cited protocol [20]. Standardized microbial suspensions were uniformly spread over Mueller-Hinton agar plates for bacteria or Sabouraud dextrose agar plates for *C. albicans*. Wells measuring 6 mm in diameter were prepared aseptically and filled with 100 µL of *D. regia* seed oil solution at 50 mg/mL in 10% (*v*/*v*) DMSO, corresponding to 5 mg of oil per well. Gentamicin and fluconazole were each tested at 1 mg/mL (100 µg per well). Because the oil and reference antimicrobial agents were applied at different amounts and may diffuse differently through the agar matrix, inhibition-zone diameters were used only as a qualitative indicator of antimicrobial activity and not as a measure of relative potency. Gentamicin (1 mg/mL) and fluconazole (1 mg/mL) were used as positive controls for the bacterial strains and *C. albicans*, respectively, while 10% DMSO served as the negative control. Bacterial plates were incubated at 37 °C for 24 h, and *C. albicans* plates were incubated at 30 °C for 48 h. Inhibition-zone diameters were measured to the nearest millimeter.

### 2.4. Determination of Minimum Inhibitory and Minimum Bactericidal or Fungicidal Concentrations

Minimum inhibitory concentrations (MIC) against the bacterial strains were determined using the broth microdilution method in 96-well plates in accordance with CLSI M07-A9 [21]. The same microdilution format was used for *C. albicans*, with RPMI 1640 serving as the growth medium. Twofold serial dilutions of the oil were prepared to obtain final test concentrations ranging from 1.95 to 1000 µg/mL. Cation-adjusted Mueller-Hinton broth was used for bacteria. The oil was initially dissolved in DMSO to prepare a stock solution. Twofold serial dilutions were subsequently prepared in the appropriate broth so that the final DMSO concentration was 1% (*v*/*v*) in the assay wells. A corresponding solvent control containing 1% (*v*/*v*) DMSO without oil was included for each microorganism to confirm that the solvent itself did not inhibit microbial growth. Each well contained 100 µL of the appropriate oil dilution and 100 µL of the microbial suspension, giving a final inoculum concentration of 5 × 10^5^ CFU/mL. Sterility controls containing oil without inoculum and growth controls containing inoculum without oil were included. Bacterial plates were incubated at 37 °C for 18–24 h, while *C. albicans* plates were incubated at 30 °C for 48 h. The MIC was defined as the lowest oil concentration at which no visible microbial growth was observed. To determine the minimum bactericidal concentration (MBC) or minimum fungicidal concentration (MFC), 10 µL aliquots from wells showing no visible growth were sub-cultured on Mueller-Hinton or Sabouraud dextrose agar, as appropriate. The MBC or MFC was defined as the lowest concentration producing a reduction of at least 99.9% in the initial viable count. Gentamicin and fluconazole were included as positive controls in the broth microdilution assays under the same inoculum, medium, temperature, and incubation conditions. Their MIC and MBC/MFC values were determined using the same procedure described for the oil.

### 2.5. Biofilm-Inhibition Assay

The effect of *D. regia* seed oil on biofilm formation was evaluated using the crystal-violet microtiter-plate assay [22]. Overnight cultures were diluted to 1 × 10^6^ CFU/mL in tryptic soy broth supplemented with 0.25% glucose. The oil was tested at concentrations corresponding to 25%, 50%, and 75% of the organism-specific MBC or MFC. The oil was prepared from the same DMSO stock solution and diluted in tryptic soy broth supplemented with 0.25% glucose to obtain the indicated test concentrations, while maintaining a final DMSO concentration of 1% (*v*/*v*) in all oil-treated wells. Corresponding solvent controls contained 1% (*v*/*v*) DMSO without oil. Untreated biofilm controls received an equal volume of 1% (*v*/*v*) DMSO without oil Each well contained the microbial inoculum and the appropriate oil concentration in a final volume of 300 µL. Wells containing growth medium alone served as blanks, while wells containing the organism without oil served as untreated biofilm controls. The plates were incubated at 37 °C for 48 h. The supernatants were then removed, and the wells were washed three times with sterile distilled water and air-dried for 30 min. Adherent biofilms were stained with 0.1% crystal violet for 15 min at room temperature. Excess stain was removed by washing the wells three times with distilled water. The retained dye was solubilized with 250 µL of 95% ethanol, and absorbance was measured at 570 nm using a BioTek 800 TS microplate reader (BioTek Instruments Inc. Winooski, VT, USA). Biofilm inhibition was calculated as follows:

$$Inhibition\left(\%\right)=1-[\frac{A_{sample}-A_{blank}}{A_{control}-A_{blank}}\times 100]$$

where *A_sample_* is the absorbance of the oil-treated biofilm, *A_control_* is the absorbance of the untreated biofilm, and *A_blank_* is the absorbance of the medium-only blank.

### 2.6. Time-Kill Kinetics

Time-kill assays were performed according to CLSI M26-A guidelines [23]. Overnight microbial cultures were diluted in fresh, organism-appropriate broth to approximately 1 × 10^6^ CFU/mL. *D. regia* seed oil was added to the previously determined MIC for each organism. Aliquots were collected after 0, 30, 60, 120, 150, and 180 min of exposure, serially diluted in sterile saline, and plated on Mueller-Hinton agar or Sabouraud dextrose agar, as appropriate. Plates were incubated at 37 °C for bacteria or 30 °C for *C. albicans* for 24–48 h, after which viable colonies were counted. The oil was added from the same DMSO stock solution to achieve the organism-specific MIC, with a final DMSO concentration of 1% (*v*/*v*). Control cultures received an equivalent concentration of DMSO without oil. Bactericidal or fungicidal activity was defined as a reduction of at least 3 log_10_ CFU/mL, corresponding to at least 99.9% killing relative to the initial inoculum.

### 2.7. DPPH Radical-Scavenging Assay

The radical-scavenging activity of *D. regia* seed oil was evaluated using the 2,2-diphenyl-1-picrylhydrazyl (DPPH) assay [24]. Oil solutions were prepared in ethanol at concentrations ranging from 1.95 to 1000 µg/mL. Oil solutions were prepared in ethanol and homogenized by vigorous vortexing for 2 min followed by sonication for 10 min. No phase separation or visible emulsion was observed. For each concentration, a sample blank was prepared identically, with ethanol replacing the DPPH solution. The absorbance of the corresponding sample blank was subtracted from the sample measurement to correct for intrinsic absorbance or turbidity of the oil. Turbidity was assessed by measuring the absorbance of the sample blank at the assay wavelength (630 nm for DPPH). In all cases, the blank absorbance was negligible (<0.05), indicating that the oil formed a homogeneous dispersion without significant interference from turbidity or light scattering.

Three milliliters of each oil solution were mixed with 1 mL of 0.1 mM DPPH prepared in ethanol. The mixtures were vortexed and incubated in the dark at room temperature for 30 min. Absorbance was subsequently measured at 630 nm using a microplate reader. Ascorbic acid served as the positive control. DPPH radical-scavenging activity was calculated using the following equation:

$$Scavenging\left(\%\right)=[\frac{A_{control}-A_{sample}}{A_{control}}]\times 100$$

where *A_control_* is the absorbance of the DPPH solution without oil and *A_sample_* is the absorbance of the reaction mixture containing the oil and it is blank-corrected. The IC_50_ was defined as the oil concentration required to scavenge 50% of the DPPH radicals.

### 2.8. ABTS Radical-Cation Scavenging Assay

The ABTS radical-cation scavenging activity of *D. regia* seed oil was determined using the cited method [25]. The ABTS radical cation was generated by reacting 7 mM 2,2′-azino-bis(3-ethylbenzothiazoline-6-sulfonic acid) with 2.45 mM potassium persulfate. The mixture was incubated in the dark at room temperature for 12–16 h. Before use, the resulting ABTS radical-cation solution was diluted with distilled water to an absorbance of 0.70 ± 0.02 at 734 nm. An aliquot of each oil solution (70 µL) was mixed with 3 mL of the diluted ABTS radical-cation solution and incubated for 6 min. Oil solutions were prepared and homogenized as described for the DPPH assay. For each concentration, a sample blank was prepared by replacing the ABTS radical-cation solution with ethanol, thereby matching the sample solvent. The absorbance of the corresponding blank was subtracted from the sample measurement before calculation of radical-scavenging activity. Blank absorbance was negligible (<0.05) at the assay wavelength. Turbidity was assessed by measuring the absorbance of the sample blank at the assay wavelength (734 nm for ABTS). In all cases, the blank absorbance was negligible (<0.05), indicating that the oil formed a homogeneous dispersion without significant interference from turbidity or light scattering. Absorbance was measured at 734 nm. Radical-cation scavenging activity was calculated using the equation described for the DPPH assay, and IC_50_ values were determined from the concentration-response curves. Ascorbic acid was used as the positive control. It was prepared in ethanol and tested over the same concentration range (1.95–1000 µg/mL) and under the same assay conditions as the oil samples. A corresponding solvent blank was included, and the IC_50_ value was determined from the concentration–response curve using the same procedure described for the DPPH assay.

### 2.9. Pancreatic Lipase-Inhibition Assay

Porcine pancreatic lipase (type II, Sigma-Aldrich; St. Louis, MO, USA) was prepared in 0.1 M potassium phosphate buffer at a final nominal activity of 200 U/mL, corresponding to 1 mg/mL protein based on the supplier-reported specific activity of 200 U/mg protein [26]. Oil concentrations ranging from 1.95 to 1000 µg/mL were preincubated with pancreatic lipase in 0.1 M potassium phosphate buffer containing 0.1% Tween 80 (pH 7.2) at 30 °C for 1 h. The reaction was initiated by adding 10 µL of a 10 mM *p*-nitrophenyl butyrate solution prepared in acetonitrile, resulting in a final reaction volume of 200 µL and a final substrate concentration of 0.5 mM. Following incubation at 30 °C for 5 min, absorbance was measured at 405 nm. Orlistat was used as the positive control. A stock solution of orlistat was prepared in DMSO and subsequently diluted with assay buffer to the required test concentrations. The final DMSO concentration was maintained at ≤1% (*v*/*v*) and was matched in the corresponding solvent-control wells. A solvent control containing the same concentration of DMSO without orlistat was included to account for any potential solvent effect. Pancreatic lipase was selected as an independent metabolic target to complement the antimicrobial and antioxidant assays within the broader bioactivity screening of *D. regia* seed oil. Pancreatic lipase inhibition was calculated as follows:

$$Inhibition\left(\%\right)=100-[\frac{B-b}{A-a}\times 100]$$

where (*A*) is the absorbance of the enzyme reaction without inhibitor, (*a*) is the absorbance of the corresponding enzyme-free control, (*B*) is the absorbance of the enzyme reaction containing the oil, and (*b*) is the absorbance of the corresponding oil-containing, enzyme-free control. IC_50_ values were calculated from the concentration-response curves.

### 2.10. Molecular Docking

#### 2.10.1. Ligand Preparation

The three-dimensional structure of stigmasterol was prepared for molecular docking using Molecular Operating Environment (MOE) software, version 2019 (Chemical Computing Group, Montreal, QC, Canada). Ligand geometry was optimized through energy minimization using the MMFF94x force field until convergence. Partial atomic charges were assigned automatically by the software, and the optimized structure was saved in the MOE database format for subsequent docking analysis [27]. Stigmasterol was selected for docking because it was one of the two most abundant phytosterols tentatively identified in the GC-MS-detectable fraction, possesses a free 3β-hydroxyl group capable of hydrogen bonding, and has previously been associated with antimicrobial and enzyme-inhibitory activities. It was therefore used as a representative phytosterol for initial target exploration rather than as the sole putative active constituent of the oil.

#### 2.10.2. Protein Preparation

The crystal structures of Sortase A from *L. monocytogenes* (PDB ID: 5HU4) and lanosterol 14α-demethylase from *C. albicans* (CYP51; PDB ID: 5V5Z) were retrieved from the RCSB Protein Data Bank (https://www.rcsb.org/). Both structures were processed using the Protein Preparation module in MOE 2019. Crystallographic water molecules and nonessential heteroatoms were removed, hydrogen atoms were added according to the assigned protonation states, and missing atom types and bond orders were corrected. The structures were then energy-minimized using the AMBER10 force field to relieve steric clashes while preserving their overall geometry.

#### 2.10.3. Binding-Site Identification

Potential ligand-binding pockets were identified using the Site Finder module in MOE 2019. Docking sites were selected on the basis of the crystal-structure topology and the location of the predicted binding cavities. Dummy atoms generated by Site Finder were used to define the docking regions.

#### 2.10.4. Docking Protocol

Stigmasterol was docked into the selected binding pocket of each protein using the Dock module in MOE 2019. Initial ligand poses were generated using the Triangle Matcher placement algorithm and ranked using the London dG scoring function. The highest-ranked poses were refined using the force-field refinement protocol with the AMBER10 force field and rescored using the GBVI/WSA dG function. For each receptor, the leading docking conformations were retained on the basis of their final docking scores, refinement root-mean-square deviation values, conformational energies, and interaction profiles.

#### 2.10.5. Protein-Ligand Interaction Analysis

The retained protein-ligand complexes were examined using the Ligand Interactions module in MOE 2019. Two-dimensional and three-dimensional interaction diagrams were generated to identify hydrogen bonds, hydrophobic contacts, and van der Waals interactions between stigmasterol and amino acid residues within each binding pocket. Interaction distances and estimated interaction energies were recorded for the best-ranked poses.

### 2.11. Statistical Analysis

All biological assays were performed in three independent biological experiments, with three technical replicates measured within each experiment. Technical replicates were used to obtain a representative value for each independent experiment, and the results are presented as the mean ± standard deviation of the three independent biological experiments, unless otherwise stated. Statistical analyses were performed using GraphPad Prism, version 8.0. IC_50_ values for DPPH radical scavenging, ABTS radical-cation scavenging, and pancreatic lipase inhibition were estimated by nonlinear regression using a four-parameter logistic model with a variable slope applied to log-transformed inhibitor concentrations and normalized response data.

## 3. Results and Discussion

### 3.1. GC-MS Profiling Reveals a Phytosterol-Rich Chemical Signature in Delonix regia Seed Oil

GC-MS profiling of the directly injected oil resulted in the tentative assignment of 18 constituents within the GC-MS-detectable fraction (Table 1 and Figure 1). The NIST match factors and characteristic mass spectral ions for all tentatively identified compounds are provided in Supplementary Table S1, allowing the confidence of each tentative assignment to be assessed. The most abundant tentatively identified compounds were β-sitosterol and stigmasterol, accounting for 26.35% and 14.57% of the total TIC peak area, respectively. Together, these two phytosterols accounted for 40.92% of the total integrated TIC peak area. Other abundant constituents included linoleic acid (10.39%), 7-methyl-*Z*-tetradecen-1-ol acetate (6.81%), and the long-chain fatty alcohol 1-heptatriacotanol (6.41%). This distribution indicates that the GC-MS-detectable fraction was enriched in phytosterol-associated peaks; however, it does not establish that phytosterols are the major components of the oil on a mass basis. Because intact triglycerides and their esterified fatty-acid components are not adequately represented by direct GC-MS without transesterification, the apparent predominance of sterols over fatty acids should not be interpreted as the absolute composition of the oil. Previous reports describing fatty-acid-dominant profiles may have analyzed derivatized or transesterified fatty-acid fractions and are therefore not directly comparable with the present analytical approach.

The abundance of β-sitosterol and stigmasterol is of biological interest because phytosterols are recognized for their cholesterol-lowering properties and have also been associated with anti-inflammatory and immunomodulatory effects [28,29]. A previous investigation quantified β-sitosterol at 310.63 mg/100 g in *D. regia* seed oil [30]. Although direct quantitative comparison is limited by differences in analytical methods and reporting units, its predominance in both studies supports β-sitosterol as a recurrent and characteristic component of the seed oil.

Among the fatty acids detected, unsaturated species were predominant, with linoleic and oleic acids accounting for 10.39% and 5.82% of the total chromatographic area, respectively. Other lipid-derived constituents included pentadecanoic acid (3.22%) and the epoxy fatty acid oxiraneoctanoic acid (4.63%). Earlier studies reported substantially higher proportions of linoleic and oleic acids in *D. regia* seed oil [31,32]. This apparent discrepancy is primarily attributable to differences in analytical methodology. Previous studies analyzed fatty-acid methyl esters following transesterification or derivatization, thereby converting esterified fatty acids into volatile derivatives suitable for GC analysis and providing a profile of the fatty-acid fraction, including fatty acids originally present in triglycerides. In contrast, the present study employed direct GC-MS analysis without derivatization, under which intact triglycerides and their esterified fatty-acid components are not adequately represented. Consequently, the relative percentages reported here describe the GC-MS-detectable fraction rather than the absolute fatty-acid or lipid composition of the oil and should not be directly compared with fatty-acid profiles obtained after FAME derivatization. Geographical origin, climatic conditions, seed maturity, and extraction procedures may also contribute to compositional differences; however, the principal explanation for the apparent discrepancy is the difference in analytical approach. Comparisons among studies should therefore consider the analytical basis used to calculate relative abundance.

The chemical profile also contained oxygenated and structurally diverse minor constituents. Oxiraneoctanoic acid and a hexanoic acid ester were detected at 4.63% and 2.86%, respectively. Such oxygenated lipid derivatives may contribute to antimicrobial or anti-inflammatory activity, although their individual contributions require experimental confirmation [33]. Additional compounds tentatively assigned included the lignan isochiapin B (1.14%), and the steroidal compound estra-1,3,5(10)-trien-17α-ol (1.52%). The coexistence of sterols, unsaturated fatty acids, esters, long-chain alcohols, and minor phenolic constituents indicates that the biological properties of the oil are more likely to arise from the combined influence of several chemical classes than from a single component.

The principal feature of the GC-MS-detectable profile in the present study was the relatively high abundance of tentatively identified β-sitosterol and stigmasterol within a broader mixture of unsaturated and oxygenated lipids. This phytosterol-enriched GC-MS fingerprint should be interpreted within the limitations of direct, non-derivatized GC-MS and does not establish absolute phytosterol predominance. Nevertheless, it provides a rationale for prioritizing β-sitosterol, stigmasterol, and linoleic acid for future targeted confirmation, quantitative analysis, and bioassay-guided fractionation. It also provides a chemical basis for interpreting the antimicrobial, antibiofilm, antioxidant, and pancreatic lipase-inhibitory activities examined in the subsequent sections.

### 3.2. Antimicrobial and Microbicidal Activities of Delonix regia Seed Oil

The antimicrobial activity of *D. regia* seed oil was evaluated against five clinically relevant pathogens using agar well diffusion and broth microdilution assays (Table 2 and Figure 2). The reference antimicrobial agents showed the expected inhibitory activity, supporting the validity of the susceptibility assays (Table 2). No direct comparison of inhibition-zone sizes between the oil and reference agents was made because they were applied at different amounts and may exhibit different diffusion characteristics in agar. The oil inhibited all tested organisms, producing inhibition zones ranging from 15 ± 0.5 mm against methicillin-resistant *Staphylococcus aureus* (MRSA) to 31 ± 0.1 mm against *Candida albicans*. The largest inhibition zones were observed against *C. albicans* and *Listeria monocytogenes*, reaching 31 ± 0.1 and 28 ± 0.7 mm, respectively. The inhibition zones produced by the oil were within the range observed for the reference antimicrobial agents; however, the oil was applied at 5 mg per well, whereas gentamicin and fluconazole were applied at 100 µg per well. Direct potency comparisons based on inhibition-zone diameters are therefore not appropriate, and the diffusion assay was used only to demonstrate antimicrobial activity. The antimicrobial activity of the oil was further evaluated using MIC and MBC/MFC values obtained by broth microdilution. The activity recorded against *Escherichia coli* and *Salmonella typhi* was consistent with previous findings for *D. regia* seed oil [34]. Inhibition-zone diameters are presented for assay validation and should not be interpreted as a direct comparison of antimicrobial potency between the oil and the reference agents because they were tested at different concentrations and may exhibit different diffusion characteristics in the agar matrix.

Broth microdilution analysis confirmed the antimicrobial activity observed in the diffusion assay. The lowest minimum inhibitory concentrations (MICs) were recorded against *L. monocytogenes*, *S. typhi*, and *C. albicans* (15.62 µg/mL), followed by *E. coli* (31.25 µg/mL) and MRSA (62.5 µg/mL). The corresponding minimum bactericidal or fungicidal concentrations were 31.25 µg/mL for *L. monocytogenes*, *S. typhi*, and *C. albicans*, 62.5 µg/mL for *E. coli*, and 125 µg/mL for MRSA. Accordingly, the MBC/MIC or MFC/MIC ratio was 2 for every tested organism, which is below the accepted threshold of 4 and is therefore consistent with bactericidal or fungicidal activity rather than growth inhibition alone [35].

The observed susceptibility pattern did not follow a simple distinction between Gram-positive and Gram-negative bacteria. Although the outer membrane of Gram-negative bacteria commonly restricts the penetration of hydrophobic antimicrobial compounds [36], *S. typhi* displayed susceptibility comparable to that of *L. monocytogenes*. This finding suggests that specific oil constituents may penetrate or destabilize the Gram-negative outer membrane or act through additional intracellular targets [37]. By contrast, MRSA was the least susceptible organism, although the oil retained measurable inhibitory and bactericidal activity against this clinically important resistant phenotype. The marked activity against *C. albicans* further demonstrates that the antimicrobial spectrum of the oil extends beyond bacterial pathogens.

The antimicrobial effects are likely to arise from the combined or complementary actions of multiple constituents rather than from a single compound. β-Sitosterol and stigmasterol, which together accounted for 40.92% of the total TIC peak area of the tentatively identified constituents, may contribute to the antimicrobial activity by altering membrane organization, fluidity, and permeability. [38]. Linoleic and oleic acids, comprising a combined 16.21%, may similarly interact with microbial lipid bilayers, promoting membrane destabilization and leakage of intracellular components [39]. The tentatively identified epoxy fatty acid and other oxygenated constituents may provide additional activity through interactions with microbial proteins, enzymes, or membrane components [40]. Minor constituents, including isochiapin B and the identified flavonoid glycoside, may also contribute through mechanisms involving nucleic acid synthesis, enzyme inhibition, or interference with quorum-sensing pathways [41]. These proposed mechanisms remain hypotheses and warrant confirmation through bioassay-guided fractionation and targeted mechanistic studies.

Overall, the convergence of agar diffusion, broth microdilution, and MBC/MFC findings demonstrates that *D. regia* seed oil exhibits antimicrobial activity against the tested panel of Gram-positive bacteria, Gram-negative bacteria, and *Candida albicans*, including methicillin-resistant *Staphylococcus aureus* (MRSA). These findings support further investigation but do not establish broad-spectrum antimicrobial activity, which would require evaluation against a substantially larger and more diverse panel of species and clinical isolates. The observed activity against taxonomically distinct microorganisms, including MRSA and *C. albicans*, provides a rationale for further fractionation of the oil, identification of the active constituents, and investigation of their individual and combined mechanisms of action. A limitation of this study is that antimicrobial activity was evaluated against only five reference microorganisms. Although the oil exhibited activity against all tested organisms, these findings cannot be generalized as evidence of broad-spectrum antimicrobial activity without testing a larger and more diverse panel of species and clinical isolates.

### 3.3. Concentration-Dependent Inhibition of Biofilm Formation by Delonix regia Seed Oil

The ability of *D. regia* seed oil to inhibit biofilm formation was evaluated against the five test organisms at concentrations corresponding to 25%, 50%, and 75% of the respective minimum bactericidal concentration (MBC) or minimum fungicidal concentration (MFC) (Table 3 and Figure 3). The oil reduced biofilm biomass in a concentration-dependent manner across all tested pathogens, although the magnitude of inhibition varied among organisms. At 75% of the MBC/MFC, inhibition approached 95%, with *Listeria monocytogenes* and *Candida albicans* each showing 94.95% inhibition. *L. monocytogenes*, *C. albicans*, and *Salmonella typhi* displayed broadly similar response patterns across the evaluated concentrations. By contrast, MRSA was substantially less responsive at 25% of the MBC, showing only 3.09% inhibition, although its susceptibility increased markedly at higher concentrations and reached 90.15% at 75% of the MBC. At the lowest concentration, *Escherichia coli* retained a comparatively strong response, with 58.21% inhibition.

The pronounced activity against *C. albicans* is particularly relevant because fungal biofilms contribute to persistent infections, reduced antifungal susceptibility, and the colonization of indwelling medical devices, especially in immunocompromised patients [42]. The ability of the oil to suppress biofilm biomass in both bacterial and fungal organisms therefore indicate activity across taxonomically distinct pathogens. Nevertheless, the lower response of MRSA at 25% of the MBC demonstrates that susceptibility remains organism- and concentration-dependent.

The observed antibiofilm activity may reflect the combined effects of several constituents identified in the oil. Phytosterols, including β-sitosterol, have been associated with reduced biofilm formation in *Staphylococcus aureus* and *Candida* species, potentially through effects on microbial adhesion, cell-surface properties, and biofilm-related gene expression [43]. Linoleic and oleic acids have also been reported to interfere with biofilm development by altering membrane properties, extracellular polymeric substance production, and quorum-sensing pathways [44]. In addition, the tentatively identified flavonoid glycoside may contribute by limiting initial cellular attachment and subsequent biofilm maturation [45]. The chemical diversity of the oil may therefore enable simultaneous interference with multiple stages of biofilm establishment.

Because the MBC/MIC and MFC/MIC ratios were 2, the concentrations corresponding to 50% and 75% of the MBC/MFC were approximately equivalent to 1 and 1.5 times the MIC, respectively. Biofilm reduction at these concentrations may consequently reflect both direct antimicrobial activity and interference with biofilm development. By contrast, the 25% MBC/MFC condition corresponded to approximately 0.5 times the MIC and therefore provides the clearest evidence of antibiofilm activity under genuinely sub-inhibitory exposure. Activity at this concentration, particularly against *E. coli* and the other responsive organisms, supports the potential of the oil to interfere with biofilm establishment before complete microbial killing occurs [46]. Moreover, the broadly comparable inhibition observed among several organisms at higher concentrations may indicate effects on conserved processes such as cellular adhesion, extracellular matrix production, or quorum-sensing regulation [47]. However, because crystal violet staining measures total attached biomass rather than microbial viability or matrix composition, these mechanisms require confirmation using complementary viability, microscopy, and gene-expression analyses.

Collectively, these findings demonstrate that *D. regia* seed oil combines planktonic antimicrobial activity with concentration-dependent suppression of biofilm formation. This dual activity across bacterial and fungal pathogens represents an important feature of the oil and provides a strong basis for bioassay-guided identification of its active constituents and further evaluation in established-biofilm and surface-associated infection models.

### 3.4. Rapid Time-Dependent Microbicidal Activity of Delonix regia Seed Oil

Time-kill analysis was performed to characterize the rate and extent of antimicrobial activity exerted by *D. regia* seed oil at the organism-specific MIC (Table 4). Viable counts decreased progressively for all tested organisms, although the killing rate varied among species. *Listeria monocytogenes* showed the most rapid response, with viable counts declining from 2.4 × 10^6^ CFU/mL at baseline to 1.75 × 10^5^ CFU/mL after 30 min and 2.14 × 10^4^ CFU/mL after 60 min. No colonies were recovered after 150 min, indicating that the viable count had fallen below the detection limit of the assay. *Escherichia coli* and *Salmonella* Typhi displayed similar time-dependent reductions, with no recoverable colonies after 180 min. MRSA exhibited a slower initial decline but likewise reached the detection limit by the final sampling point. A comparable response was recorded for *Candida albicans*, demonstrating that the rapid microbicidal effect of the oil extended to both bacterial and fungal organisms. These findings indicate reductions in viable counts to below the assay detection limit, corresponding to ≥3 log_10_ (≥99.9%) reductions relative to the initial inoculum, and should not be interpreted as absolute or theoretical 100% killing.

The observed endpoint reductions met the ≥3 log_10_ (≥99.9%) threshold commonly used to characterize microbicidal activity, further supporting the microbicidal profile indicated by the MBC/MIC and MFC/MIC ratios [35]. Importantly, time-kill analysis complements these endpoint measurements by demonstrating that microbial killing occurred rapidly and progressively rather than merely reflecting growth inhibition at the end of the incubation period. Rapid reduction in the viable microbial population may help restrict the opportunity for persistence and subsequent biofilm establishment, although the clinical relevance of this effect requires confirmation under physiologically representative conditions [48].

Differences in killing rates likely reflect organism-specific variations in cell-envelope architecture, membrane composition, stress-response pathways, and the ability to tolerate membrane-active compounds. The slower initial response of MRSA compared with *L. monocytogenes* may therefore arise from its distinctive envelope properties and antimicrobial defense mechanisms rather than from peptidoglycan thickness alone [49]. Nevertheless, the eventual reduction in MRSA to below the detection limit indicates that these protective mechanisms did not prevent the terminal antimicrobial effect of the oil.

Collectively, the kinetic findings extend the MIC and MBC/MFC results by demonstrating rapid, time-dependent reductions in viable counts to below the assay detection limit. across Gram-positive bacteria, Gram-negative bacteria, and yeast. This broad microbicidal behavior strengthens the evidence that *D. regia* seed oil contains constituents capable of acting against structurally diverse microbial targets and supports further fractionation and mechanistic investigation of its active components.

### 3.5. Concentration-Dependent Radical-Scavenging Activity of Delonix regia Seed Oil

The radical-scavenging capacity of *D. regia* seed oil was evaluated using the complementary DPPH (Figure 4) and ABTS assays (Figure 5). The oil produced a clear concentration-dependent response in both assays. DPPH radical scavenging increased from 23.8% at 1.95 µg/mL to 93.1% at 1000 µg/mL, whereas ABTS radical scavenging increased from 29.0% to 90.0% over the same concentration range. The corresponding IC_50_ values were 17.04 ± 0.25 µg/mL for DPPH and 13.17 ± 0.40 µg/mL for ABTS. As expected, ascorbic acid was more potent than the oil, with IC_50_ values of 7.12 ± 0.03 and 3.77 ± 0.04 µg/mL in the DPPH and ABTS assays, respectively.

The lower IC_50_ value obtained using the ABTS assay indicates that the oil constituents reacted more efficiently under the conditions of this assay. This difference should not be attributed exclusively to greater sensitivity toward lipophilic antioxidants, because ABTS can detect both hydrophilic and lipophilic radical-scavenging compounds. Differences between the two assays may instead reflect variations in radical structure, solvent compatibility, reaction kinetics, and the accessibility of individual oil constituents to the radical species [50]. The consistent concentration-dependent responses obtained using both methods reduce the likelihood that the observed activity was specific to a single assay system.

The radical-scavenging activity may arise from the combined contributions of several constituents. Phytosterols, including β-sitosterol and stigmasterol, possess hydroxyl groups and have been associated with radical-scavenging and redox-modulating effects [51]. The tentatively identified flavonoid glycoside and isochiapin B may also contribute through electron or hydrogen donation and metal-binding mechanisms commonly associated with phenolic compounds [52]. However, the relative contribution of each compound cannot be established from assays performed on the whole oil. Fatty acid composition may influence the overall oxidative stability of the oil, although linoleic acid is susceptible to lipid peroxidation and should not be considered a principal antioxidant constituent [53]. Oxygenated lipid derivatives, including the tentatively assigned oxirane-containing compound, may further influence the redox behavior of the oil, but their contribution requires direct experimental confirmation [40].

Although the DPPH IC_50_ obtained for *D. regia* seed oil appears lower than values reported for several edible oils [54], comparisons among independent studies should be made cautiously because IC_50_ values are strongly influenced by extraction procedures, solvent systems, radical concentrations, incubation periods, and calculation methods. The present findings nevertheless demonstrate notable radical-scavenging capacity under the standardized conditions employed in this study. Further fractionation, oxidative-stability testing, and cell-based studies will be required to identify the principal active constituents and determine whether the observed chemical antioxidant activity translates into protection in biological or food systems.

Overall, the concordant DPPH and ABTS results establish *D. regia* seed oil as a concentration-dependent source of radical-scavenging constituents. This dual-assay evidence strengthens the multifunctional biological profile of the oil and provides a compelling basis for investigating its active fractions as potential natural antioxidant ingredients.

### 3.6. Concentration-Dependent Pancreatic Lipase Inhibition by Delonix regia Seed Oil

The pancreatic lipase inhibitory activity of *D. regia* seed oil was evaluated using *p*-nitrophenyl butyrate as the substrate (Figure 6). This assay was included as a metabolic endpoint independent of the antimicrobial and antioxidant evaluations, to determine whether the same oil also exhibited inhibitory activity against pancreatic lipase, an established target involved in dietary fat digestion and obesity-related metabolic intervention. The oil produced a clear concentration-dependent response, with inhibition increasing from 14.6% at 1.95 µg/mL to 92.6% at 1000 µg/mL. Its IC_50_ value was 26.29 ± 0.08 µg/mL, compared with 5.30 ± 0.01 µg/mL for orlistat, the clinically used reference inhibitor. Although the oil was approximately fivefold less potent than orlistat based on the IC_50_ values, its defined concentration-response relationship demonstrates substantial enzyme-inhibitory activity for unfractionated natural oil.

The IC_50_ value obtained in this study is within the range reported for several plant-derived pancreatic lipase inhibitors [55]. Nevertheless, comparisons among studies should be interpreted cautiously because apparent inhibitory potency is influenced by the enzyme source, substrate type and concentration, buffer composition, incubation conditions, and data-analysis method. The present findings therefore establish the activity of the oil under the specified experimental conditions but do not demonstrate equivalence to purified inhibitors evaluated using different assay systems.

The observed response likely reflects the collective contribution of multiple constituents rather than the action of a single compound. Phytosterols, including β-sitosterol and stigmasterol, have been associated with pancreatic lipase inhibition and may also influence lipid-water interfacial properties that are essential for enzyme activity [56]. Fatty acids may modify apparent lipase activity through product-mediated feedback, interactions with the enzyme, or changes in substrate emulsification [57]. Oxygenated lipids and esterified compounds could similarly affect substrate accessibility or interact with residues within or near the catalytic pocket [58]. However, the present data does not demonstrate direct or covalent binding to the catalytic serine residue. The tentatively identified flavonoid glycoside may also contribute because flavonoids can interact with pancreatic lipase through hydrogen bonding and hydrophobic contacts [59]. Bioassay-guided fractionation, enzyme-kinetic analysis, and targeted molecular docking would be required to identify the active constituents and determine their modes of inhibition.

The physiological relevance of these findings should be considered carefully. The use of *p*-nitrophenyl butyrate provides an efficient screening model but does not fully reproduce the interfacial hydrolysis of dietary long-chain triglycerides in the presence of bile salts, colipase, and other gastrointestinal components. Moreover, an in vitro enzyme assay alone cannot establish anti-obesity efficacy or prevention of metabolic syndrome. Confirmation using physiologically relevant lipid substrates, simulated gastrointestinal conditions, and appropriate in vivo models is therefore necessary before potential weight-management applications can be proposed [60]. Moreover, the pharmacokinetic behavior and formulation requirements of a highly lipophilic oil must also be considered. Systemic delivery could be limited by poor aqueous solubility, gastrointestinal digestion and first-pass metabolism following oral administration, extensive protein or lipid binding, and uncertain tissue distribution. Therefore, the present in vitro findings should not be extrapolated to systemic infections. Topical or localized delivery may represent a more feasible direction for future investigation for accessible skin, wound, mucosal, or device-associated infections and biofilms, where direct exposure to locally effective concentrations may be more achievable. However, this possibility remains speculative and requires dedicated formulation, penetration, safety, pharmacokinetic, and in vivo efficacy studies.

Overall, the concentration-dependent inhibition of pancreatic lipase adds a metabolic enzyme target to the antimicrobial, antibiofilm, and antioxidant activities demonstrated by *D. regia* seed oil. This integrated biological profile provides a strong rationale for identifying the constituents responsible for lipase inhibition and evaluating their activity under more physiologically representative conditions.

### 3.7. Comparative Docking of Stigmasterol with Listeria monocytogenes Sortase A and Candida albicans CYP51

Molecular docking was employed to examine the predicted binding of stigmasterol, one of the most abundant phytosterols tentatively identified in the GC-MS-detectable fraction of *Delonix regia* seed oil, to two pathogen-associated molecular targets. Stigmasterol was selected as a representative phytosterol based on its relative abundance in the GC-MS-detectable fraction, structural features relevant to ligand–protein interactions, and previous reports of antimicrobial and enzyme-inhibitory properties. The docking results are exploratory and are not intended to attribute the biological activity of the whole oil to this single compound. Docking can provide useful hypotheses regarding ligand orientation and molecular recognition; however, docking scores are not experimentally determined binding affinities and should not be interpreted as direct evidence of biological activity or complex stability [27].

Stigmasterol was accommodated within the predicted binding pocket of *L. monocytogenes* Sortase A (PDB ID: 5HU4), with the five retained poses producing docking scores (S) ranging from −6.60 to −6.04 kcal/mol (Table 5). The top-ranked pose yielded an S-score of −6.60 kcal/mol and an RMSD refinement value of 0.79 Å. The hydroxyl group of stigmasterol formed a hydrogen bond with MET63 at a distance of 3.26 Å and an estimated interaction energy of −1.1 kcal/mol (Table 6). The tetracyclic sterol scaffold occupied a predominantly hydrophobic region of the pocket, indicating that van der Waals and hydrophobic contacts contributed substantially to the predicted ligand accommodation (Figure 7). The RMSD refinement value describes the displacement of the pose during computational refinement and should not, in isolation, be regarded as evidence of experimentally validated binding stability.

Sortase A is a membrane-associated cysteine transpeptidase that anchors LPXTG-containing surface proteins to the peptidoglycan of Gram-positive bacteria. These surface proteins participate in adhesion, host-cell invasion, immune evasion, and biofilm development, making Sortase A an attractive target for antivirulence strategies [61]. Nevertheless, the reported stigmasterol pose was characterized principally by an interaction with MET63 and did not show direct contact with the established catalytic triad of *L. monocytogenes* Sortase A. The docking result should therefore be interpreted as evidence of predicted accommodation within a putative binding pocket rather than proof of catalytic-site inhibition. Enzyme-inhibition and site-directed interaction studies are required to establish whether stigmasterol modulates Sortase A activity.

Docking against *C. albicans* CYP51 (PDB ID: 5V5Z) generated less favorable scores under the same computational protocol. The five retained poses produced S scores ranging from −3.41 to −1.14 kcal/mol, and the top-ranked pose had an S-score of −3.41 kcal/mol and an RMSD refinement value of 1.04 Å (Table 6). In this pose, the hydroxyl oxygen of stigmasterol formed a hydrogen bond with ARG381 at a distance of 2.68 Å and an estimated interaction energy of −0.5 kcal/mol (Table 7). The remaining stabilization was attributed primarily to hydrophobic contacts between the sterol nucleus and nonpolar residues lining the predicted binding cavity (Figure 8).

CYP51 is a heme-containing cytochrome P450 enzyme that catalyzes lanosterol 14α-demethylation during ergosterol biosynthesis. Because ergosterol is essential for fungal membrane structure and function, CYP51 is a principal target of azole antifungal agents [62]. Unlike azoles, stigmasterol lacks a heterocyclic nitrogen atom capable of coordinating directly with the heme iron. The predicted pose was therefore dominated by hydrophobic contacts and a single hydrogen bond rather than heme coordination, which may partly account for its less favorable score. This finding suggests that stigmasterol is unlikely to reproduce the characteristic binding mode of conventional azole inhibitors.

Within the limitations of cross-target docking comparisons, the more negative score obtained for the Sortase A complex indicates more favorable predicted accommodation of stigmasterol in that pocket than in the CYP51 pocket under the present scoring protocol. This difference should not be described as definitive target selectivity because docking scores are influenced by receptor structure, pocket size, flexibility, and scoring-function behavior. Moreover, the antimicrobial activity of the whole oil cannot be attributed to stigmasterol alone. The oil contains multiple tentatively identified constituents that may contribute individually or collectively to the observed biological activities. The predicted stigmasterol-target interactions should therefore be regarded as testable mechanistic hypotheses rather than a complete explanation for the activity of the oil.

Overall, the docking analysis provides a testable mechanistic hypothesis linking a major phytosterol in *D. regia* seed oil to pathogen-associated protein targets. The results prioritize the predicted stigmasterol-Sortase A interaction for experimental evaluation while indicating comparatively limited compatibility with the CYP51 catalytic environment. This integration of chemical profiling, biological assays, and target-oriented modeling strengthens the mechanistic scope of the study without implying that computational predictions establish causality.

### 3.8. Integrated Composition-Activity Relationships and Mechanistic Interpretation

The multifunctional biological profile of *Delonix regia* seed oil can be interpreted in relation to its complex chemical composition. A hypothesis-based overview linking the principal constituent classes tentatively identified by GC-MS with the antimicrobial, antibiofilm, antioxidant, and pancreatic lipase-inhibitory activities is presented in Figure 9. The connections shown are based on published literature and previously proposed mechanisms associated with these compound classes and were not experimentally established in the present study. The arrows and associations do not represent direct experimental links between individual constituents and specific biological effects. Rather, they are inferred from previous reports concerning phytosterols, unsaturated fatty acids, phenolic compounds, and other oxygenated lipids. No individual compound was isolated or tested independently, and possible additive or synergistic interactions were not evaluated. The framework should therefore be regarded as hypothesis-generating and is intended to guide future bioassay-guided fractionation and mechanistic validation.

Phytosterols represented the most abundant tentatively identified class within the GC-MS-detectable fraction, with β-sitosterol and stigmasterol collectively accounting for 40.92% of the total chromatographic peak area. Their amphiphilic structures, comprising a rigid hydrophobic sterol nucleus, a flexible side chain, and a single hydroxyl group, permit interactions with lipid membranes, hydrophobic protein pockets, and reactive chemical species [63]. These properties may contribute to the antimicrobial and antibiofilm effects of the oil through alterations in microbial membrane organization and surface-associated processes. Phytosterols may also participate in radical-scavenging and enzyme-binding interactions; however, the present experiments do not demonstrate that they are individually responsible for the observed antioxidant or lipase inhibitory activities. The docking results provide additional evidence that stigmasterol can be accommodated within pathogen-associated protein pockets, particularly the selected pocket of *L. monocytogenes* Sortase A, but do not establish target inhibition or a direct causal relationship with the antimicrobial findings.

Linoleic and oleic acids accounted for 10.39% and 5.82% of the total TIC peak area, respectively. Unsaturated fatty acids can affect microbial membrane permeability, surface adhesion, extracellular matrix production, and quorum-sensing-regulated biofilm development [44]. Their amphiphilic properties may also influence substrate emulsification and interfacial behavior in the pancreatic lipase assay. By contrast, their contribution to the measured radical-scavenging activity is likely limited, particularly because polyunsaturated linoleic acid is susceptible to lipid peroxidation. The antioxidant response is therefore more plausibly associated with phytosterols and minor redox-active constituents than with the unsaturated fatty acids alone.

The tentatively identified flavonoid glycoside and isochiapin B, representing 2.84% and 1.14%, respectively, may contribute disproportionately to the radical-scavenging response because phenolic structures can participate in electron donation, hydrogen-atom transfer, and metal binding [64]. These constituents may also complement the antimicrobial and antibiofilm activities through effects on microbial enzymes, adhesion, or cell-signaling pathways. Although additive or synergistic interactions between major lipophilic constituents and minor phenolic compounds are plausible [65], synergy was not directly evaluated and should not be inferred without testing isolated fractions, reconstructed mixtures, or defined compound combinations.

Other oxygenated constituents may further influence the activity of the oil. Epoxide-containing molecules can display electrophilic reactivity toward biological nucleophiles [66]; however, the presence of an oxirane group alone does not demonstrate covalent enzyme inhibition. Accordingly, the tentatively assigned oxiraneoctanoic acid cannot currently be identified as the source of pancreatic lipase inhibition or antimicrobial activity. Long-chain alcohols and esters may modify the physicochemical behavior of the oil, including its interactions with microbial membranes, proteins, and assay substrates [67], but their individual biological contributions remain unresolved.

The tentative identification of ethyl iso-allocholate is noteworthy because bile acid derivatives have been associated with antimicrobial and metabolic regulatory properties [68]. Nevertheless, the occurrence of this compound in a plant seed oil is unusual, and its identity should be confirmed using an authentic standard or an orthogonal analytical method before it is included in the mechanistic interpretation. This caution also applies to other low-abundance or structurally unusual GC-MS assignments.

Taken together, the findings support a multicomponent model in which abundant phytosterols and fatty acids establish the principal lipophilic matrix, while minor oxygenated and phenolic-type constituents may broaden or modulate its biological activity. Such multifunctionality is characteristic of chemically complex natural products and may involve additive, complementary, or synergistic effects across several molecular and cellular processes [69]. However, demonstrating polypharmacology requires direct evaluation of individual compounds, active fractions, and defined combinations.

Overall, integrating tentative GC-MS profiling with multiple biological assays and target-oriented docking provides a provisional framework for prioritizing constituents within the GC-MS-detectable fraction of *D. regia* seed oil for further investigation. The principal novelty of the study lies in combining tentative chemical profiling with measured antimicrobial, antibiofilm, antioxidant, and pancreatic lipase-inhibitory activities, while generating experimentally testable molecular hypotheses for subsequent validation. Because no individual compound was definitively identified or absolutely quantified, the proposed composition-activity associations should be regarded as provisional and hypothesis-generating rather than established. Further bioassay-guided fractionation, compound isolation, confirmation using authentic standards, absolute quantification, and direct target-validation studies will be required before any structure–activity relationship can be established.

## 4. Conclusions

This study identifies a phytosterol-enriched, GC-MS-detectable fraction of *Delonix regia* seed oil with antimicrobial, antibiofilm, antioxidant, and pancreatic lipase-inhibitory activities in vitro. The most abundant tentatively identified GC-MS peaks were β-sitosterol and stigmasterol, accompanied by unsaturated fatty acids and oxygenated compounds. These assignments remain tentative, and no absolute quantification of individual constituents was performed. The oil inhibited clinically relevant bacterial and fungal pathogens and suppressed biofilm formation. Time-kill analysis demonstrated rapid microbicidal activity, characterized by ≥3 log_10_ CFU/mL reductions with no recoverable colonies within 3 h under the experimental conditions used. The oil also exhibited radical-scavenging and pancreatic lipase-inhibitory effects. These findings indicate that different chemical classes may contribute collectively to the observed biological activities, although the roles of individual constituents and their possible interactions remain to be established. In particular, the tentative assignment of a polar flavonoid glycoside in the nonpolar *n*-hexane extract is analytically and chemically unexpected, requires independent confirmation, and should be interpreted with caution. Molecular docking provided exploratory evidence for potential stigmasterol interactions with *Listeria monocytogenes* Sortase A and *Candida albicans* CYP51; however, these predictions require biochemical and mechanistic validation. Importantly, the present findings are limited to in vitro experiments and should not be interpreted as evidence of clinical efficacy. Further studies involving compound confirmation and quantification, bioassay-guided fractionation, safety assessment, formulation development, pharmacokinetic evaluation, and appropriate in vivo models are required before any potential practical or therapeutic application can be considered.

## 5. Limitation and Future Perspectives

This study has several important limitations. The antimicrobial, antibiofilm, antioxidant, and pancreatic lipase-inhibitory activities were demonstrated only under the specific in vitro conditions used in this study and do not establish therapeutic efficacy in vivo. GC-MS assignments were tentative, and no absolute quantification of individual compounds was performed; therefore, the reported TIC peak-area percentages should not be interpreted as absolute concentrations or mass proportions. Another limitation is that residual *n*-hexane was not quantitatively assessed. Although the solvent was removed under reduced pressure and the recovered oil was subsequently flushed with nitrogen, complete removal was not independently verified. No *n*-hexane peak was identified among the reported GC-MS constituents; however, the GC-MS method used was not designed or validated as a residual-solvent assay, and trace residual amounts therefore cannot be excluded. Dedicated residual-solvent analysis, such as validated headspace GC, would be required to confirm its absence or quantify any residual *n*-hexane and to determine whether it could influence the observed biological activities. In addition, cytotoxicity, hemolytic activity, irritation potential, systemic toxicity, bioavailability, pharmacokinetics, biodistribution, and tissue penetration were not evaluated. The lipophilic nature of the oil and the concentrations required for antimicrobial activity may also present important formulation and delivery challenges. The mechanism of action remains unresolved, and the composition-activity relationships proposed here should be considered hypothesis-generating. Likewise, the docking analysis was restricted to stigmasterol as a representative tentatively identified phytosterol. This does not imply that stigmasterol alone accounts for the biological activity of the whole oil; rather, the analysis provides a hypothesis-generating starting point for future mechanistic investigation. Future studies should include confirmation and absolute quantification of individual constituents using authentic standards, validated residual-solvent analysis, bioassay-guided fractionation, mechanistic experiments, comprehensive safety assessment, appropriate formulation strategies, pharmacokinetic evaluation, and in vivo studies before any therapeutic relevance can be established. Molecular modeling was limited to stigmasterol as a representative phytosterol. β-Sitosterol, despite its large relative GC-MS peak area, was not modeled. The modeling results therefore cannot be generalized to all major constituents of the oil.

## Figures and Tables

**Figure 1:**
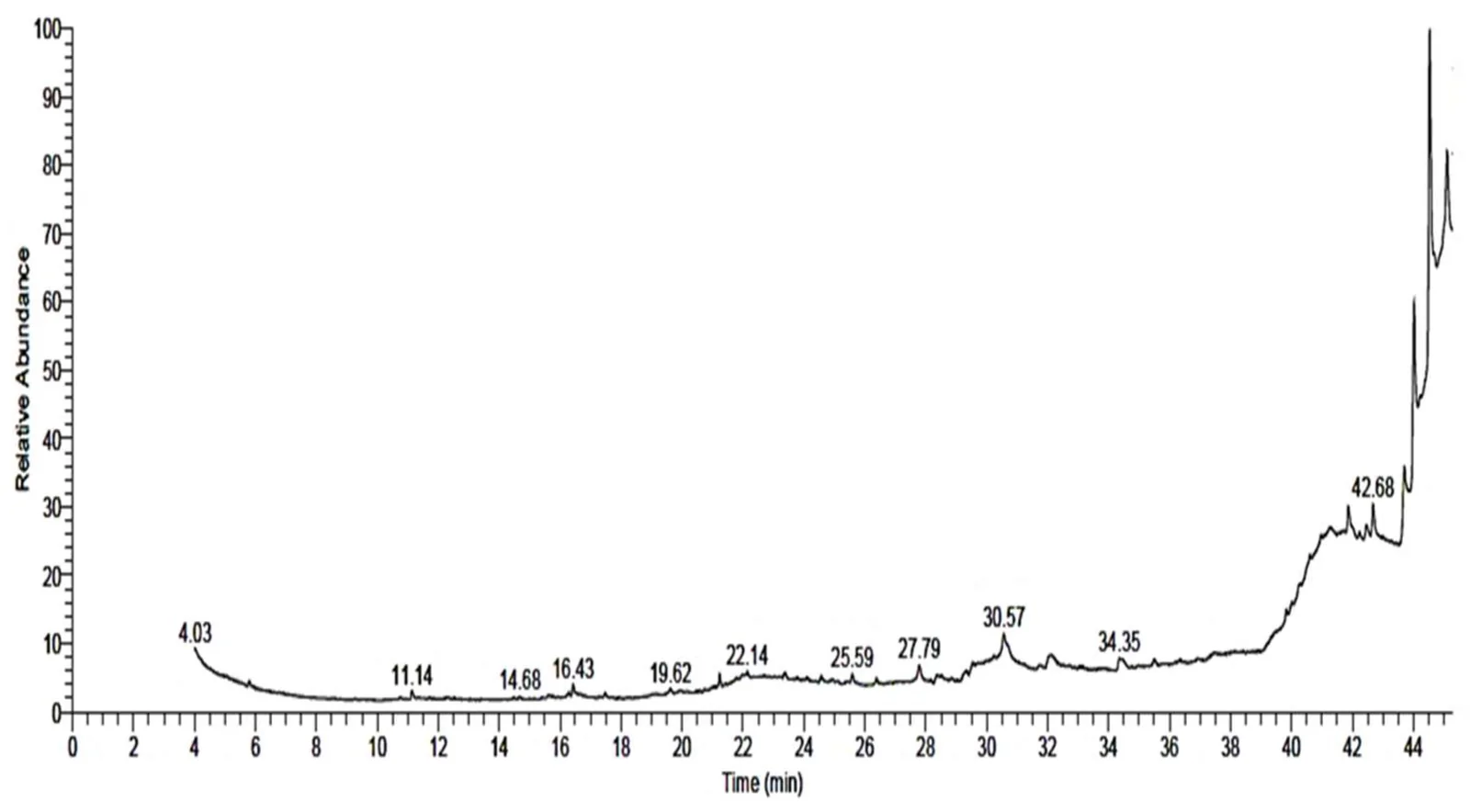
Total ion chromatogram (TIC) of *Delonix regia* seed oil analyzed by gas chromatography-mass spectrometry (GC-MS) using an HP-5MS capillary column. Major chromatographic peaks are labeled with the names and retention times of the tentatively identified constituents. The predominant peaks were assigned to β-sitosterol, stigmasterol, linoleic acid, 7-methyl-*Z*-tetradecen-1-ol acetate, and 1-heptatriacotanol. Peak assignments are tentative, and relative peak areas are not quantitative and should not be interpreted as representing the absolute composition of the oil.

**Figure 2:**
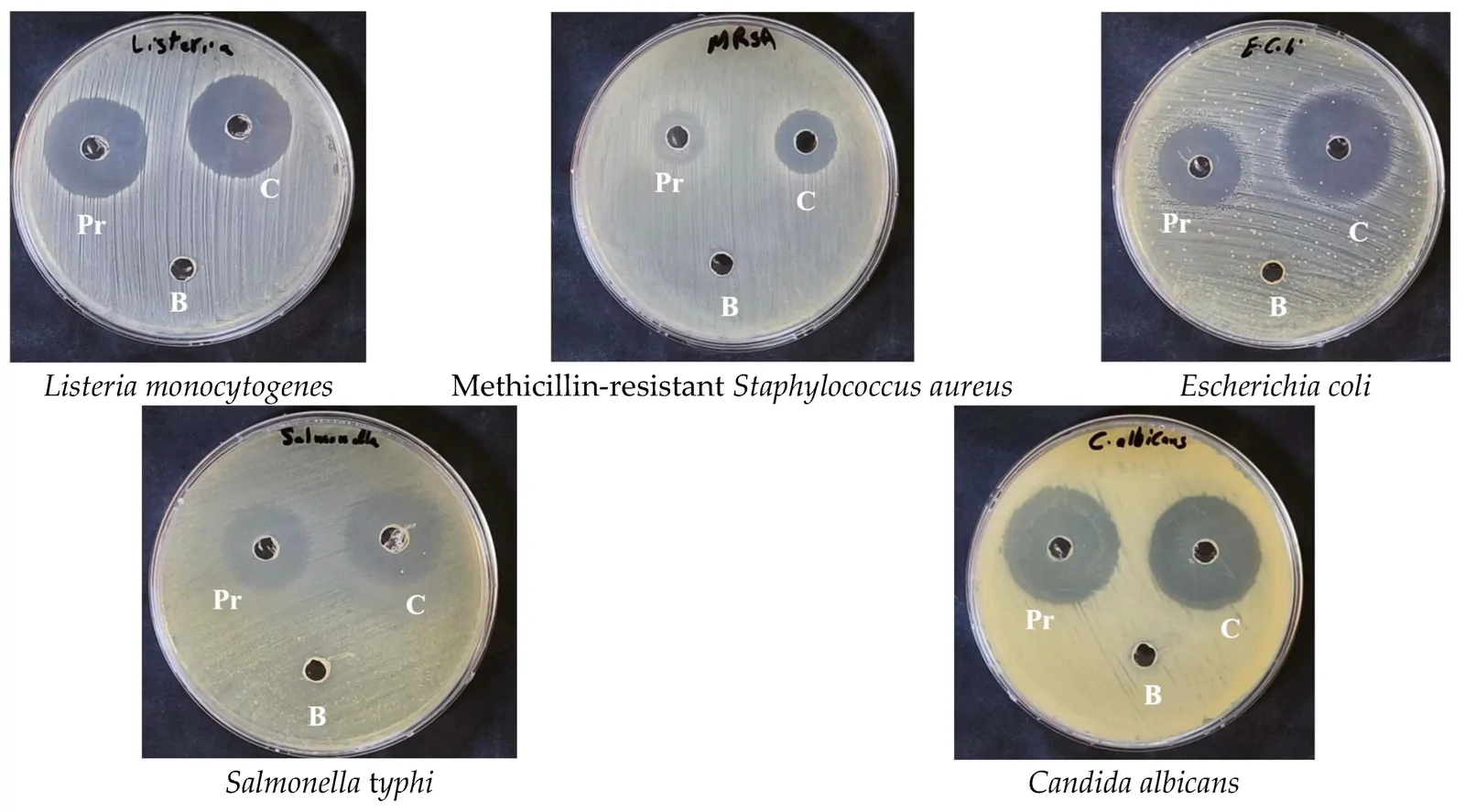
In vitro antimicrobial activity of *Delonix regia* seed oil (50 mg/mL) against five clinically relevant pathogens, expressed as inhibition-zone diameters (mm). Gentamicin (1 mg/mL) and fluconazole (1 mg/mL) were used as positive controls for bacterial strains and *Candida albicans*, respectively, whereas 10% DMSO served as the vehicle control. Values are presented as the mean ± SD of three independent biological experiments, each performed with three technical replicates (*n* = 9 measurements), Pr, *D. regia* seed oil; B, vehicle blank (10% DMSO); C, corresponding positive control.

**Figure 3:**
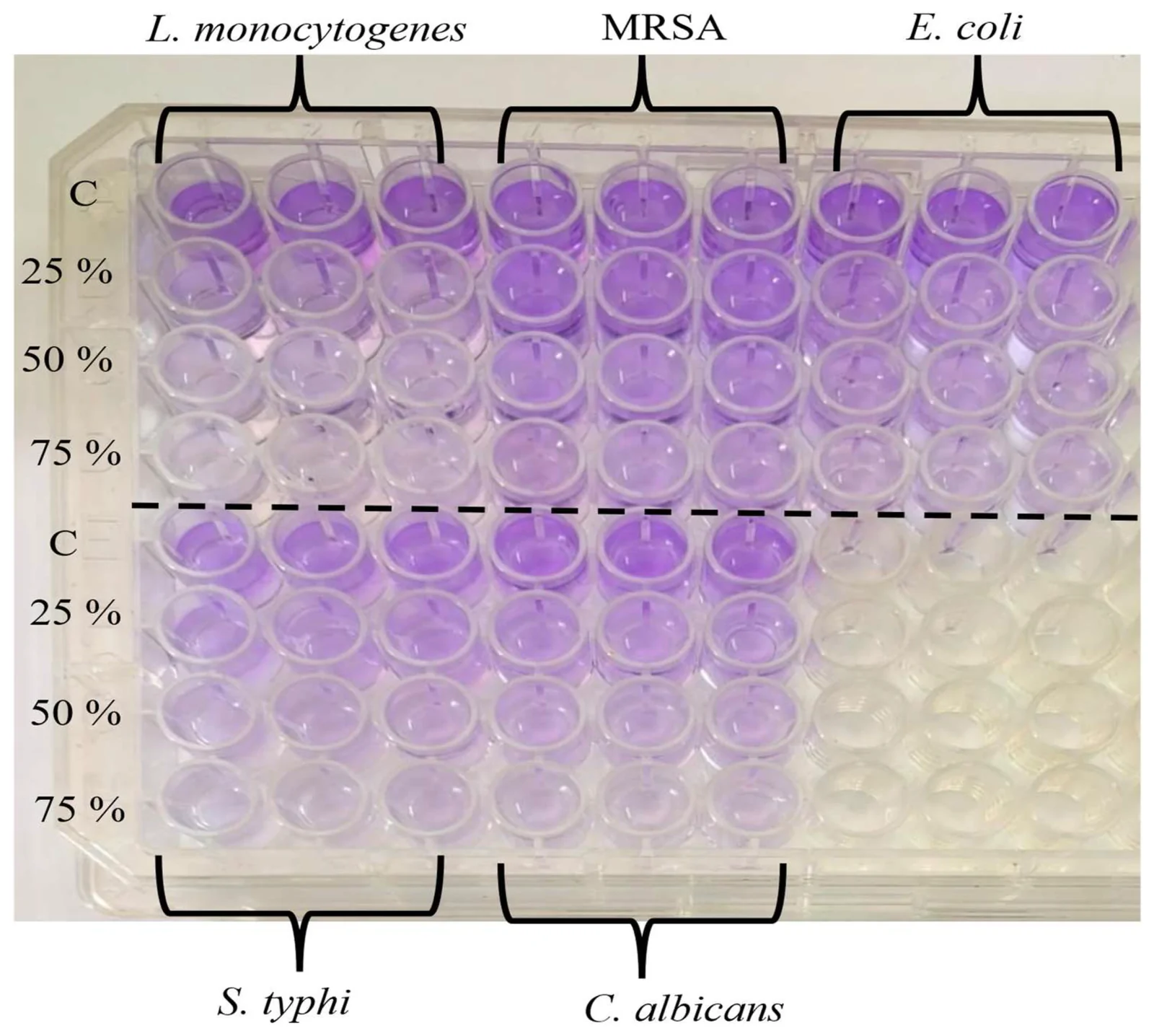
Concentration-dependent effects of *Delonix regia* seed oil on biofilm formation by five clinically relevant pathogens. The oil was evaluated at 25%, 50%, and 75% of the organism-specific minimum bactericidal concentration (MBC) for bacterial strains or minimum fungicidal concentration (MFC) for *Candida albicans*. Attached biofilm biomass was quantified using the crystal violet microtiter plate assay, and inhibition was calculated relative to the untreated organism control. Values are presented as the mean ± SD of three independent biological experiments, each performed with three technical replicates (*n* = 9 measurements).

**Figure 4:**
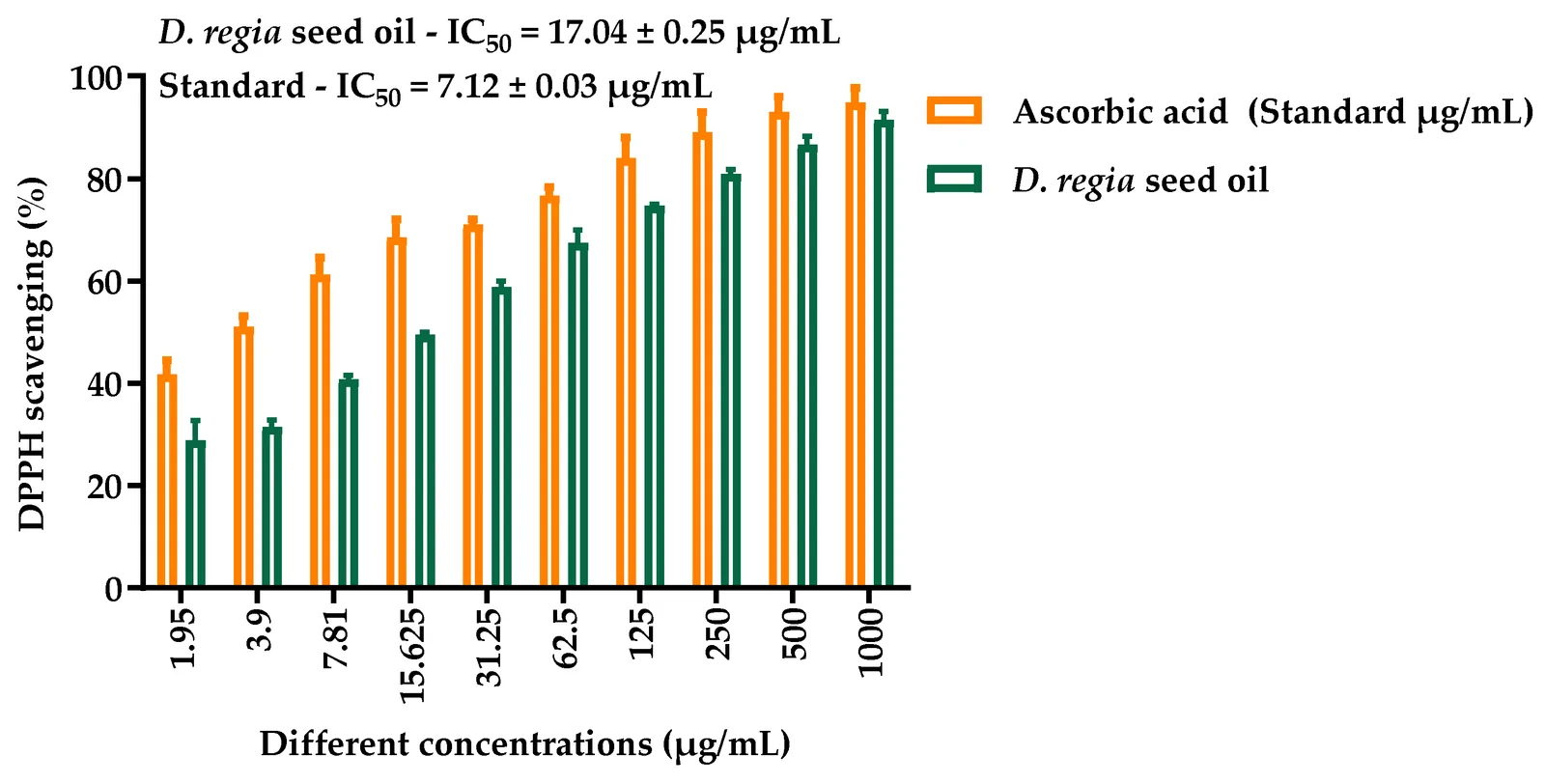
DPPH radical-scavenging activity of *Delonix regia* seed oil compared with ascorbic acid as the positive control. Bars represent IC_50_ values, defined as the concentration required to scavenge 50% of DPPH radicals; a lower IC_50_ value indicates greater radical-scavenging activity. The IC_50_ values were 17.04 ± 0.25 µg/mL for the seed oil and 7.12 ± 0.03 µg/mL for ascorbic acid. Data are presented as the mean ± SD of three independent biological experiments, each performed in triplicate (*n* = 9 measurements). Sample blanks were prepared at each concentration and subtracted to correct for intrinsic absorbance or turbidity.

**Figure 5:**
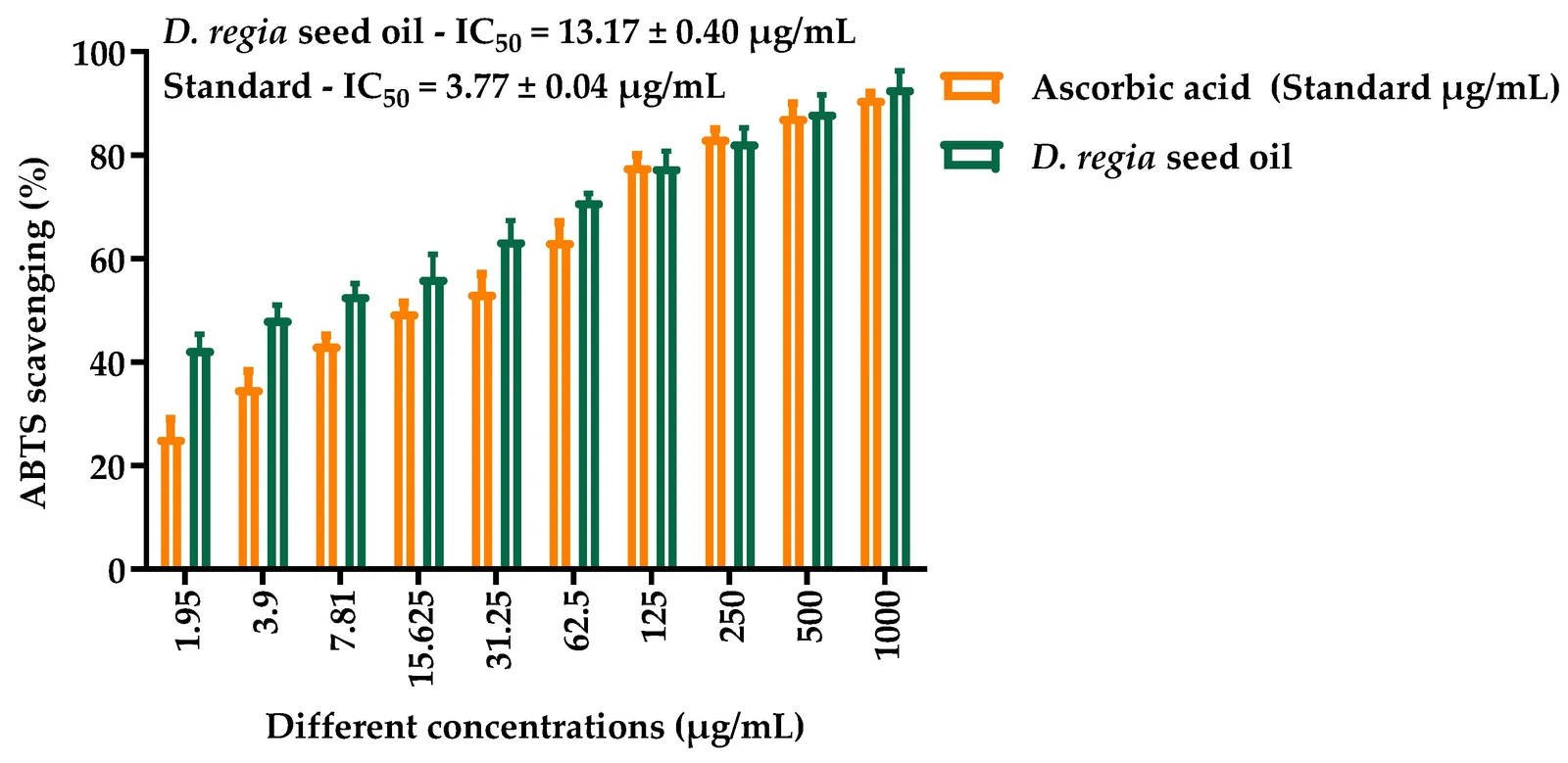
ABTS radical cation-scavenging activity of *Delonix regia* seed oil compared with ascorbic acid as the positive control. Bars represent IC_50_ values, defined as the concentration required to scavenge 50% of ABTS radical cations; a lower IC_50_ value indicates greater radical-scavenging activity. The IC_50_ values were 13.17 ± 0.40 µg/mL for the seed oil and 3.77 ± 0.04 µg/mL for ascorbic acid. Data are presented as the mean ± SD of three independent biological experiments, each performed with three technical replicates (*n* = 9 measurements). Sample blanks were prepared at each concentration and subtracted from the corresponding sample measurements to correct for intrinsic absorbance or turbidity.

**Figure 6:**
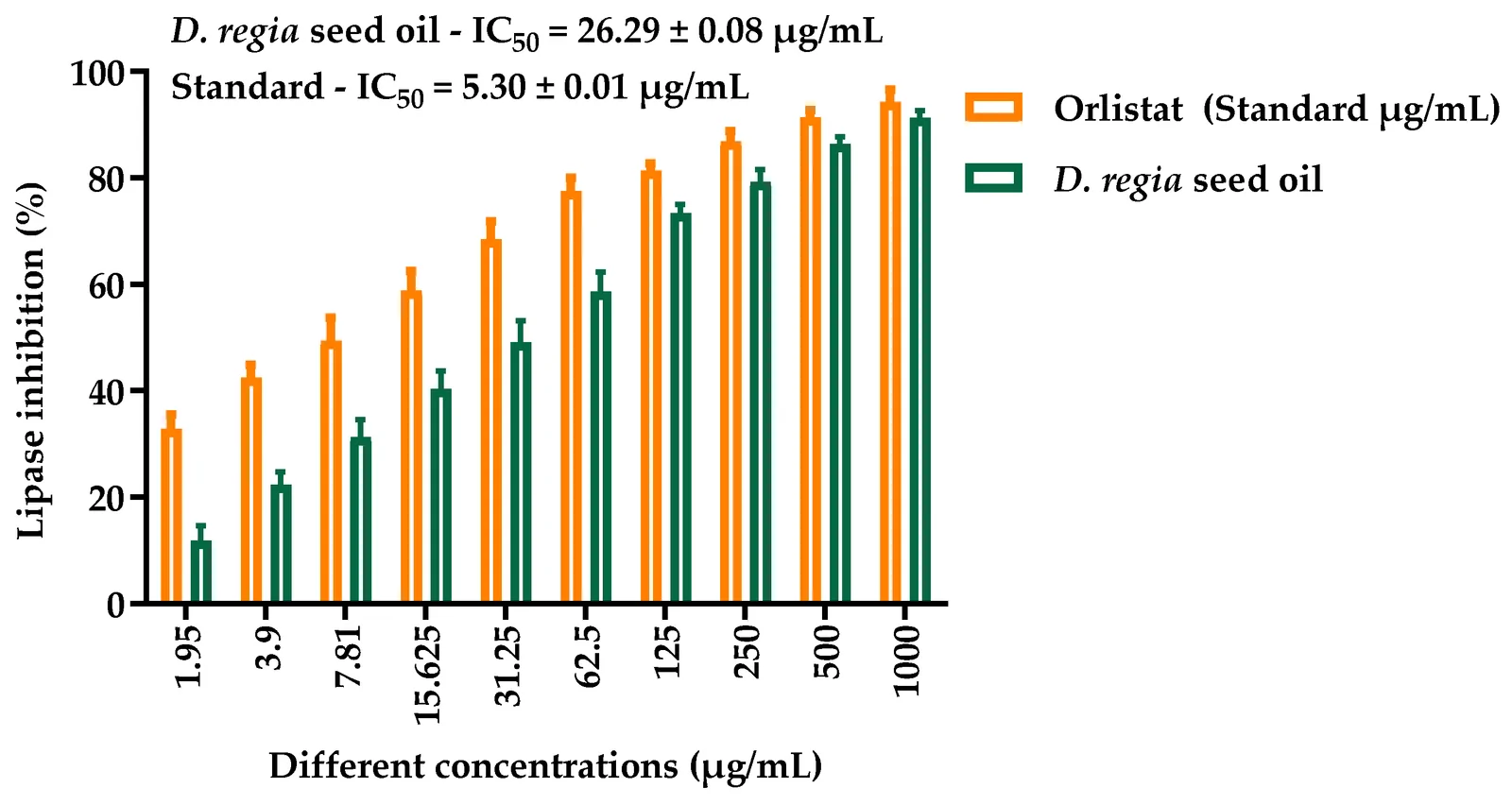
Pancreatic lipase inhibitory activity of *Delonix regia* seed oil compared with orlistat as the positive control. Porcine pancreatic lipase activity was measured using *p*-nitrophenyl butyrate as the substrate. Bars represent the IC_50_ value, defined as the concentration required to reduce enzyme activity by 50%; a lower IC_50_ indicates greater inhibitory potency. The IC_50_ values were 26.29 ± 0.08 µg/mL for seed oil and 5.30 ± 0.01 µg/mL for orlistat. Values are presented as the mean ± SD of three independent biological experiments, each performed with three technical replicates (*n* = 9 measurements). Orlistat was dissolved in DMSO and subsequently diluted in assay buffer; the final DMSO concentration was ≤1% (*v*/*v*) and was matched in the corresponding solvent controls. Pancreatic lipase concentration is reported as enzymatic activity (U/mL).

**Figure 7:**
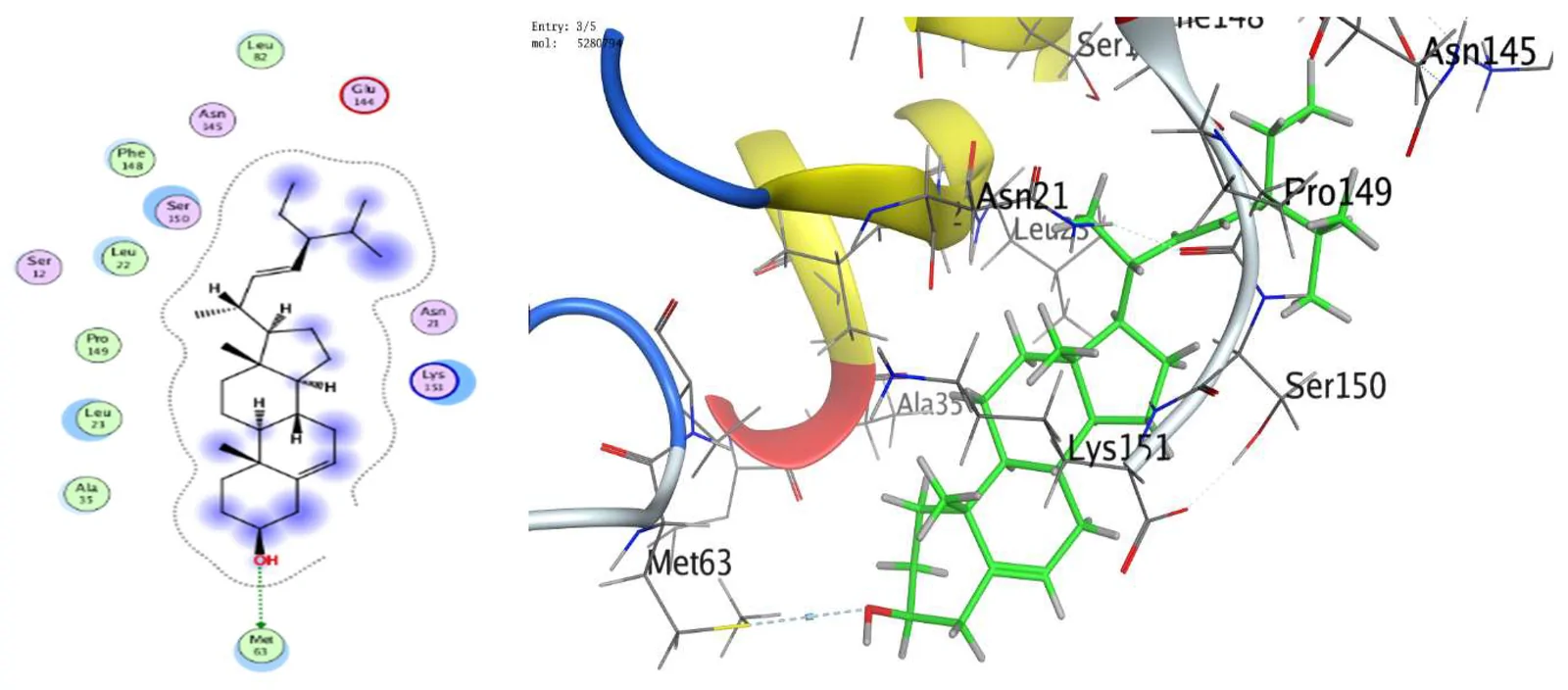
Predicted binding mode of stigmasterol within the selected binding pocket of Sortase A from *Listeria monocytogenes* (PDB ID: 5HU4). The two-dimensional interaction map shows the hydrogen bond formed with MET63 and the surrounding noncovalent contacts, whereas the three-dimensional representation illustrates the orientation of the top-ranked stigmasterol pose within the protein pocket.

**Figure 8:**
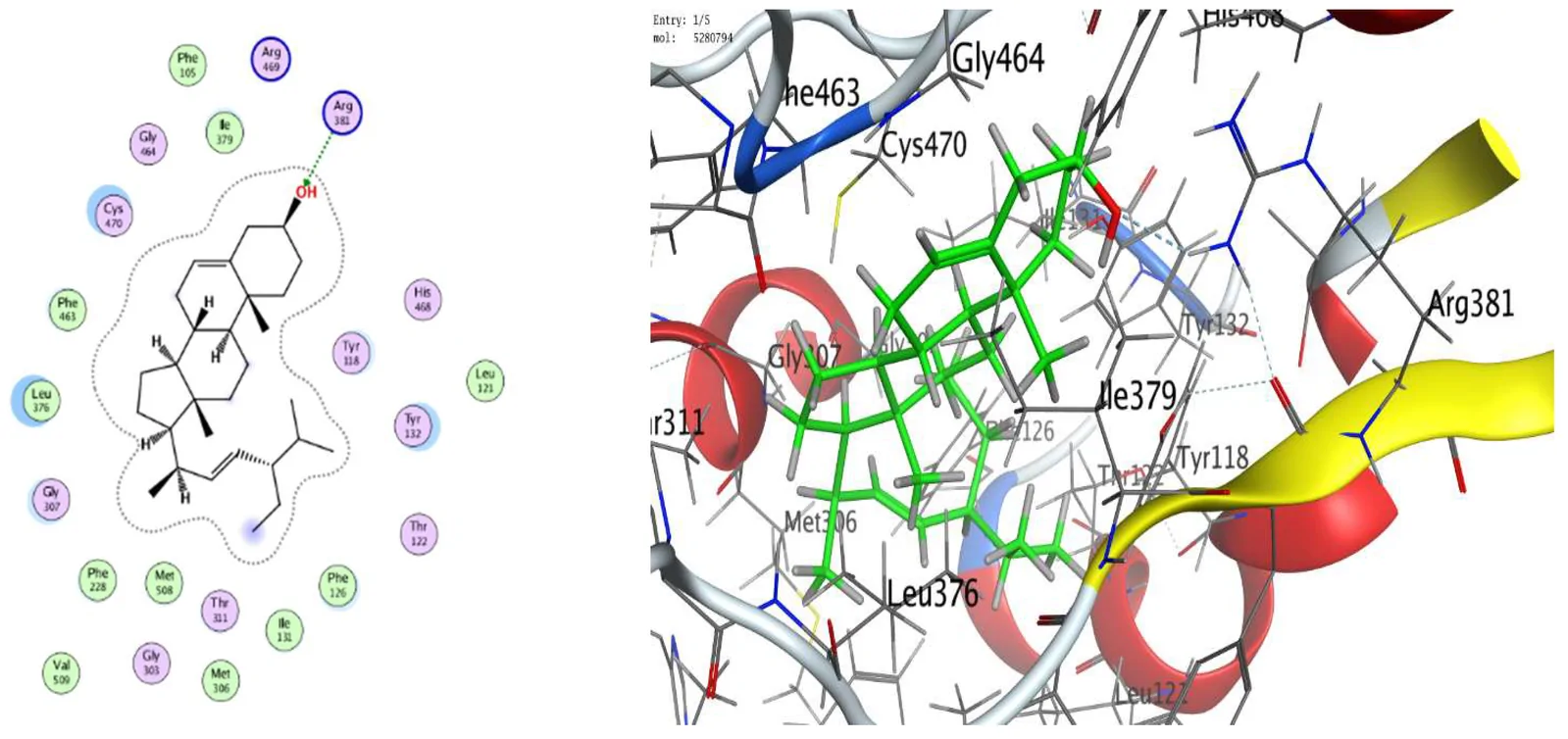
Predicted binding mode of stigmasterol within the selected binding pocket of lanosterol 14α-demethylase (CYP51) from *Candida albicans* (PDB ID: 5V5Z). The two-dimensional interaction map shows the hydrogen bond formed with ARG381 and the surrounding noncovalent contacts, whereas the three-dimensional representation illustrates the orientation of the top-ranked stigmasterol pose within the CYP51 binding cavity.

**Figure 9:**
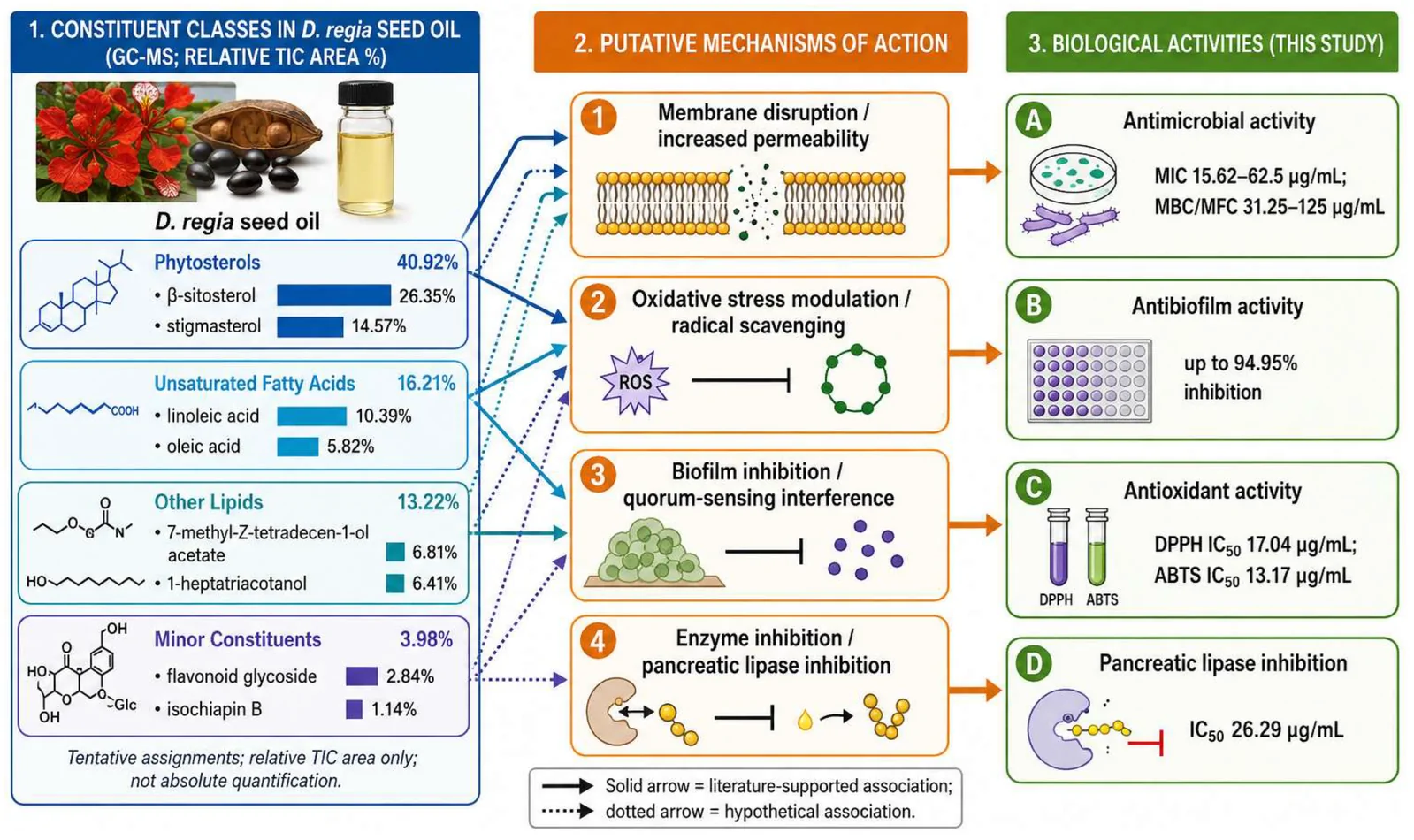
Integrated composition–activity framework for *Delonix regia* seed oil. The left panel summarizes the principal constituent classes tentatively identified by GC-MS, the central panel illustrates plausible literature-supported biological processes, and the right panel presents the antimicrobial, antibiofilm, radical-scavenging, and pancreatic lipase-inhibitory activities evaluated experimentally in the present study. Connections between constituent classes, proposed mechanisms, and biological effects are literature-based, hypothesis-generating associations and were not established experimentally in this study. They should not be interpreted as evidence of the activity, mechanism, or synergistic contribution of any individual constituent. The figure is intended to guide future fractionation and mechanistic validation rather than to present an established structure–activity relationship.

**Table 1:** Constituents tentatively identified in *Delonix regia* seed oil by GC-MS, with their retention times, chemical classes, molecular formulas, molecular weights and relative peak-area percentages.

Compound Name	RT (min)	* Relative TIC Area (%)	Class	Molecular Formula	Molecular Weight
1-Hexadecanol, 2-methyl-	5.80	0.55	Fatty alcohol	C_17_H_36_O	256
Dotriacontane	15.62	2.85	Alkane	C_32_H_66_	450
Tetradecane, 2,6,10-trimethyl-	16.43	2.00	Alkane	C_17_H_36_	240
Hexanoic acid, 2-ethyl-, oxybis(2,1-ethanediyloxy-2,1-ethanediyl) ester	21.98	2.86	Ester	C_24_H_46_O_7_	446
2-[5-(2-Hydroxy-propyl)-tetrahydrofuran-2-yl]-propionic acid, t-butyl ester	22.15	1.75	Ester	C_14_H_26_O_4_	258
Pentadecanoic acid	28.51	3.22	Fatty acid	C_15_H_30_O_2_	242
Hexadecanoic acid, 2,3-dihydroxypropyl ester	29.29	2.00	Ester (glyceryl ester)	C_19_H_38_O_4_	330
Isochiapin B	29.54	1.14	Lignan	C_19_H_22_O_6_	346
Oxiraneoctanoic acid, 3-octyl-, cis-	30.56	4.63	Epoxy fatty acid	C_18_H_34_O_3_	298
Estra-1,3,5(10)-trien-17á-ol	31.72	1.52	Steroid	C_18_H_24_O	256
7-Methyl-Z-tetradecen-1-ol acetate	32.04	6.81	Ester	C_17_H_32_O_2_	268
9-Octadecenoic acid (Z)-	37.44	5.82	Fatty acid	C_18_H_34_O_2_	282
Ethyl iso-allocholate	41.26	4.29	Ester (bile acid ester)	C_26_H_44_O_5_	436
1-Heptatriacotanol	43.70	6.41	Fatty alcohol	C_37_H_76_O	536
Stigmasterol	44.02	14.57	Sterol	C_29_H_48_O	412
β-Sitosterol	44.53	26.35	Sterol	C_29_H_50_O	414
9,12-Octadecadienoic acid (Z, Z)-	45.11	10.39	Fatty acid	C_18_H_32_O_2_	280

* No internal standard or calibration curve was used. Values are relative total-ion-chromatogram (TIC) peak-area percentages and do not represent absolute concentrations. Relative abundance was calculated from TIC peak areas. Compound assignments were based primarily on comparison of the acquired mass spectra with the NIST 2017 mass-spectral library, with published retention-index values included as supportive information where available. Because an *n*-alkane reference series was not analyzed, experimental linear retention indices were not calculated; therefore, the literature retention-index values should not be interpreted as definitive confirmation of compound identity. All compound assignments remain tentative.

**Table 2:** Antimicrobial activity of *Delonix regia* seed oil against five clinically relevant pathogens, assessed using agar well diffusion and broth microdilution assays. Inhibition-zone diameters are presented as the mean ± SD of three replicate assays (*n* = 3). MIC, minimum inhibitory concentration; MBC, minimum bactericidal concentration; MFC, minimum fungicidal concentration.

Microorganism	Inhibition Zone (mm)		MIC, Oil (µg/mL)	MBC/MFC, Oil (µg/mL)	MIC, Positive Control (µg/mL)	MBC/MFC, Positive Control (µg/mL)
	Oil	* Positive Control				
*Listeria monocytogenes* ATCC 19115	28 ± 0.7	26 ± 0.5	15.62	31.25	1.0	2.0
Methicillin-resistant *Staphylococcus aureus* ATCC 33591	15 ± 0.5	17 ± 0.3	62.5	125	4.0	8.0
*Escherichia coli* ATCC 8739	22 ± 0.5	29 ± 0.5	31.25	62.5	1.0	2.0
*Salmonella typhi* ATCC 6539	21 ± 0.3	24 ± 0.7	15.62	31.25	1.0	2.0
*Candida albicans* ATCC 10221	31 ± 0.1	29 ± 0.5	15.62	31.25	0.5	1.0

* Values are presented as the mean ± SD of three replicate assays (*n* = 3). Gentamicin was used as the positive control for bacterial strains and fluconazole for *C. albicans*. For agar well diffusion, the oil was tested at 50 mg/mL and the positive controls at 1 mg/mL. For broth microdilution, the positive controls were tested in parallel under the same experimental conditions as the oil. Direct potency comparisons between the oil and reference antimicrobial agents based on inhibition-zone diameters are not appropriate because of differences in test concentrations and diffusion characteristics.

**Table 3:** Inhibitory effects of *Delonix regia* seed oil on biofilm formation by five clinically relevant pathogens at 25%, 50%, and 75% of the organism-specific minimum bactericidal concentration (MBC) or minimum fungicidal concentration (MFC). Biofilm inhibition was determined using the crystal violet microtiter plate assay and is expressed as the mean percentage inhibition ± SD of three independent biological experiments, each performed in triplicate (*n* = 9 measurements).

Microorganism	Antibiofilm Activity at		
	75% MBC	50% MBC	25% MBC
*Listeria monocytogenes* ATCC 19115	94.95%	83.54%	76.06%
Methicillin-resistant *Staphylococcus aureus* ATCC 33591	90.15%	69.75%	3.09%
*Escherichia coli* ATCC 8739	93.17%	82.85%	58.21%
*Salmonella typhi* ATCC 6539	95.48%	84.13%	75.46%
*Candida albicans* ATCC 10221	94.95%	83.54%	76.06%

**Table 4:** Time-kill kinetics of *Delonix regia* seed oil at the organism-specific minimum inhibitory concentration (MIC) against selected pathogens during a 180-min exposure period. Viable microbial counts are expressed as CFU/mL at the indicated sampling times. A value of zero indicates that no colonies were recovered and that the viable count was below the detection limit of the assay.

Time Point	Viable Cell Count (CFU/mL)—Mean ± SD			
	*Listeria monocytogenes*	Methicillin-Resistant *Staphylococcus aureus* ATCC 33591	*Escherichia coli* ATCC 8739	*Salmonella typhi* ATCC 6539
0	24 × 10^5^ ± 1.0	17 × 10^5^ ± 2.0	25 × 10^5^ ± 0.9	31 × 10^5^ ± 2.4
30 min	175 × 10^3^ ± 1.0	88 × 10^4^ ± 1.4	151 × 10^4^ ± 0.6	29 × 10^4^ ± 1.2
1 h	214 × 10 ± 1.1	52 × 10^2^ ± 1.2	39 × 10^3^ ± 1.4	23 × 10^3^ ± 1.5
2 h	25	62 × 10 ± 1.0	121 × 10^2^ ± 1.0	116 × 10 ± 1.5
2.5 h	0	33	161	111
3 h	0	0	0	0

Data are presented as the mean viable count ± SD from three independent biological experiments, each performed with three technical replicates (*n* = 9 measurements). The oil was evaluated at the previously determined organism-specific MICs, which ranged from 15.62 to 62.5 µg/mL. At 180 min, no colonies were recovered from any of the tested organisms; therefore, values reported as 0 CFU/mL represent viable counts below the assay detection limit. This outcome corresponded to a ≥3 log_10_ (≥99.9%) reduction relative to the initial inoculum. For the bacterial strains, this reduction met the criterion for bactericidal activity described in the CLSI M26-A guideline. CFU, colony-forming units; MIC, minimum inhibitory concentration; SD, standard deviation.

**Table 5:** Molecular Operating Environment docking results for the five top-ranked poses of stigmasterol within the predicted binding pocket of *Listeria monocytogenes* Sortase A (PDB ID: 5HU4). The table presents the final docking score (S), RMSD refinement value, and associated energy parameters generated during pose placement, refinement, and rescoring. More negative S-scores indicate more favorable predicted ligand-receptor interactions within the applied scoring protocol.

Mol	S	rmsd_refine	E_conf	E_place	E_score1	E_refine	E_score2
Stigmasterol	−6.60245	0.79571682	−43.5116	−72.3537	−8.63602	−30.8891	−6.60245
Stigmasterol	−6.22761	1.9074864	−58.0727	−50.7931	−8.78343	−27.2489	−6.22761
Stigmasterol	−6.18622	1.9211564	−47.3344	−74.957	−9.29522	−28.0475	−6.18622
Stigmasterol	−6.10919	1.6572537	−45.8673	−72.6229	−9.75601	−27.9005	−6.10919
Stigmasterol	−6.03789	1.3965583	−56.6685	−49.6888	−8.65836	−25.9743	−6.03789

**Table 6:** Molecular Operating Environment docking results for the five top-ranked poses of stigmasterol within the predicted binding pocket of CYP51 from *Candida albicans* (PDB ID: 5V5Z). The table presents the final docking score (S), RMSD refinement value, and associated energy parameters generated during pose placement, refinement, and rescoring. More negative S-scores indicate more favorable predicted ligand-receptor interactions within the applied scoring protocol.

Mol	S	rmsd_refine	E_conf	E_place	E_score1	E_refine	E_score2
Stigmasterol	−3.40689	1.0429116	−149.539	−84.4507	−10.627	−40.83686	−3.40689
Stigmasterol	−2.57894	1.3838468	−155.331	−64.7423	−8.13076	−51.9759	−2.57894
Stigmasterol	−2.42292	1.2830355	−180.468	−64.6458	−9.60623	−56.33134	−2.42292
Stigmasterol	−1.67127	1.8642083	−177.884	−64.7879	−8.92489	−67.20467	−1.67127
Stigmasterol	−1.13823	1.5681272	−172.776	−63.722	−7.66807	−70.26288	−1.13823

**Table 7:** Predicted molecular interactions of stigmasterol with Sortase A from *Listeria monocytogenes* (PDB ID: 5HU4) and CYP51 from *Candida albicans* (PDB ID: 5V5Z). Interacting residues, interaction types, distances, and estimated interaction energies are reported for the top-ranked docking pose of each complex.

Mol	Protein	Ligand	Receptor	Interaction	Distance	E (kcal/mol)
Stigmasterol	5HU4	O1 1	SD MET 63 (A)	H-donor	3.26	−1.1
	5V5Z	O1 1	NH1 ARG 381 (A)	H-acceptor	2.68	−0.5

## Data Availability

Data that supports the findings of this study are available within the article and from the corresponding author upon request.

## Supplementary Materials

The following supporting information can be downloaded at: https://www.mdpi.com/article/10.3390/antiox15101249/s1. Table S1. Full mass spectral data for compounds tentatively identified in Delonix regia seed oil by GC-MS, including retention time, relative TIC peak area, chemical class, molecular formula, molecular weight, characteristic mass spectral ions, and NIST match factor. The molecular weight of estra-1,3,5(10)-trien-17a-ol (C18H24O2) is 272.39 g/mol. Therefore, the value of 256 reported in the original table appears inconsistent with the molecular formula and should be verified and corrected. No internal standard or calibration curve was used. Values are expressed as relative TIC peak-area percentages and do not represent absolute concentrations. Compound assignments were based on comparisons with the NIST 2017 mass spectral library and published retention-index data. Characteristic mass spectral ions (*m*/*z*) and NIST match factors (%) are provided for each tentatively identified compound. NIST match factors represent spectral similarity scores and do not constitute definitive compound identification; assignments with match factors below 90% should therefore be considered particularly tentative. No authentic reference standards were analyzed.
