# Supplementary material for: Phytosterol-Rich Delonix regia Seed Oil Exhibits Antimicrobial, Antibiofilm, Radical-Scavenging and Pancreatic Lipase-Inhibitory Activities: Integrated GC-MS Profiling and Molecular Docking of Stigmasterol
Source: Antioxidants (Basel). 2026 Sep 28;15(10):1249. doi: 10.3390/antiox15101249 (PMC13700883; doi:10.3390/antiox15101249)
Supplement: Supplementary file 1 [file antioxidants-15-01249-s001.zip › antioxidants-4547602-supplementary.pdf]

Article

Phytosterol-Rich *Delonix regia* Seed Oil Exhibits Antimicrobial, Antibiofilm, Radical-Scavenging and Pancreatic Lipase-Inhibitory Activities: Integrated GC-MS Profiling and Molecular Docking of Stigmasterol

Husam Qanash [1,2,*], Aisha M. H. Al-Rajhi [3], Abdulrahman S. Bazaid [1,2], Fahad Almarshadi [4], Walid Alesefir [5], Abdu Aldarhami [6], Waleed Hakami [7], Amro Duhduh [7] and Abeer Omar Ahmed [8]

[1] Department of Medical Laboratory Science, College of Applied Medical Sciences, University of Ha'il, Hail 55476, Saudi Arabia
[2] Medical and Diagnostic Research Center, University of Ha'il, Hail 55473, Saudi Arabia
[3] Department of Biology, College of Science, Princess Nourah bint Abdulrahman University, P.O. Box 84428, Riyadh 11671, Saudi Arabia
[4] Public Health Department, College of Public Health and Health Informatics, University of Ha'il, Hail 55473, Saudi Arabia
[5] Department of Internal Medicine, College of Medicine, University of Ha'il, Hail 55473, Saudi Arabia
[6] Department of Microbiology and Parasitology, Faculty of Medicine, Umm Al-Qura University, Al-Qunfudah 28815, Saudi Arabia
[7] Department of Medical Laboratory Technology, College of Nursing and Health Sciences, Jazan University, Jazan 45142, Saudi Arabia
[8] Department of Pharmaceutics, College of Pharmacy, Jazan University, Jazan 45142, Saudi Arabia
* Correspondence: h.qanash@uoh.edu.sa

Table S1. Full mass spectral data for compounds tentatively identified in *Delonix regia* seed oil by GC-MS, including retention time, relative TIC peak area, chemical class, molecular formula, molecular weight, characteristic mass spectral ions, and NIST match factor.

No.	Compound Name	RT (min)	Relative TIC area (%)	Class	Molecular Formula	Molecular Weight	Characteristic ions (m/z)	NIST match (%)
**1**	1-Hexadecanol, 2-methyl-	5.80	0.55	Fatty alcohol	C17H36O	256	256 [M]+, 241, 227, 213, 199, 185, 171, 157, 143, 129, 111, 97, 83, 69, 57	91
**2**	Dotriacontane	15.62	2.85	Alkane	C32H66	450	450 [M]+, 422, 394, 366, 338,	95

							310, 282, 254, 226, 198, 170, 142, 114, 85, 71, 57	
**3**	Tetradecane, 2,6,10-trimethyl-	16.43	2.00	Alkane	$C_{17}H_{36}$	240	240 [M]+, 225, 211, 197, 183, 169, 155, 141, 127, 113, 99, 85, 71, 57	93
**4**	Hexanoic acid, 2-ethyl-, oxybis(2,1-ethanedioxy-2,1-ethanediy) ester	21.98	2.86	Ester	$C_{24}H_{46}O_7$	446	446 [M]+, 431, 413, 385, 357, 329, 301, 273, 245, 217, 189, 161, 133, 105, 77, 57	87
**5**	2-[5-(2-Hydroxy-propyl)-tetrahydrofuran-2-yl]-propionic acid, t-butyl ester	22.15	1.75	Ester	$C_{14}H_{26}O_4$	258	258 [M]+, 243, 225, 202, 185, 169, 157, 143, 129, 115, 101, 87, 73, 57	85
**6**	Pentadecanoic acid	28.51	3.22	Fatty acid	$C_{15}H_{30}O_2$	242	242 [M]+, 227, 213, 199, 185, 171, 157, 143, 129, 115, 101, 87, 73, 60, 55	96
**7**	Hexadecanoic acid, 2,3-dihydroxypropyl ester	29.29	2.00	Ester (glyceryl ester)	$C_{19}H_{38}O_4$	330	330 [M]+, 312, 299, 281, 267, 253, 239, 225, 211, 197, 183, 169, 155, 141, 127, 113, 99, 85, 71, 57	89
**8**	Isochiapin B	29.54	1.14	Lignan	$C_{19}H_{22}O_6$	346	346 [M]+, 331, 313, 303, 285, 267, 257, 239, 221, 203, 185, 167, 149, 131, 103, 77	82
**9**	Oxiraneoctanoic acid, 3-octyl-, cis-	30.56	4.63	Epoxy fatty acid	$C_{18}H_{34}O_3$	298	298 [M]+, 280, 265, 251, 237, 223, 209, 195, 181, 167, 153, 139, 125, 111, 97, 83, 69, 55	88

**10**	Estra-1,3,5(10)-trien-17a-ol	31.72	1.52	Steroid	C18H24O2	256*	256 [M]+, 241, 238, 223, 213, 199, 185, 171, 157, 143, 129, 115, 105, 91, 77, 55	84
**11**	7-Methyl-Z-tetradecen-1-ol acetate	32.04	6.81	Ester	C17H32O2	268	268 [M]+, 253, 236, 225, 208, 193, 179, 166, 151, 138, 123, 110, 95, 82, 69, 55	92
**12**	9-Octadecenoic acid (Z)- (Oleic acid)	37.44	5.82	Fatty acid	C18H34O2	282	282 [M]+, 264, 239, 222, 207, 194, 180, 166, 152, 137, 123, 111, 97, 83, 69, 55	97
**13**	Ethyl iso-allocholate	41.26	4.29	Ester (bile acid ester)	C26H44O5	436	436 [M]+, 421, 403, 385, 371, 357, 343, 329, 315, 301, 287, 273, 259, 245, 231, 217, 203, 189, 175, 161, 147, 133, 119, 105, 91, 77, 55	80
**14**	4H-1-Benzopyran-4-one, 2-(3,4-dihydroxyphenyl)-6,8-di-α-D-glucopyranosyl-5,7-dihydroxy	42.68	2.84	Flavonoid glycoside	C27H30O16	610	610 [M]+, 592, 574, 448, 430, 412, 394, 376, 358, 340, 322, 304, 286, 268, 250, 232, 214, 196, 178, 160, 142, 124, 106, 88, 60	78
**15**	1-Heptatriacotanol	43.70	6.41	Fatty alcohol	C37H76O	536	536 [M]+, 521, 503, 475, 448, 420, 392, 364, 336, 308, 280, 252, 224, 196, 168, 140, 112, 97, 83, 69, 57	94
**16**	Stigmasterol	44.02	14.57	Sterol	C29H48O	412	412 [M]+, 394, 379, 351, 300, 271, 255, 213,	98

							159, 145, 133, 119, 105, 91, 83, 69, 55	
**17**	β-Sitosterol	44.53	26.35	Sterol	C29H50O	414	414 [M]+, 396, 381, 329, 303, 273, 255, 213, 161, 145, 107, 95, 81, 69, 55	99
**18**	9,12-Octadecadienoic acid (Z,Z)-(Linoleic acid)	45.11	10.39	Fatty acid	C18H32O2	280	280 [M]+, 262, 237, 220, 205, 194, 180, 164, 150, 137, 123, 109, 95, 81, 67, 55	97

The molecular weight of estra-1,3,5(10)-trien-17a-ol ($C_{18}H_{24}O_2$) is 272.39 g/mol. Therefore, the value of 256 reported in the original table appears inconsistent with the molecular formula and should be verified and corrected. No internal standard or calibration curve was used. Values are expressed as relative TIC peak-area percentages and do not represent absolute concentrations. Compound assignments were based on comparisons with the NIST 2017 mass spectral library and published retention-index data. Characteristic mass spectral ions (m/z) and NIST match factors (%) are provided for each tentatively identified compound. NIST match factors represent spectral similarity scores and do not constitute definitive compound identification; assignments with match factors below 90% should therefore be considered particularly tentative. No authentic reference standards were analyzed.
